# Study of the anticancer effect of new quinazolinone hydrazine derivatives as receptor tyrosine kinase inhibitors

**DOI:** 10.3389/fchem.2022.969559

**Published:** 2022-11-17

**Authors:** Motahareh Mortazavi, Masoumeh Divar, Tahereh Damghani, Fatemeh Moosavi, Luciano Saso, Somayeh Pirhadi, Mehdi Khoshneviszadeh, Najmeh Edraki, Omidreza Firuzi

**Affiliations:** ^1^ Medicinal and Natural Products Chemistry Research Center, Shiraz University of Medical Sciences, Shiraz, Iran; ^2^ Department of Physiology and Pharmacology “Vittorio Erspamer”, Sapienza University of Rome, Rome, Italy; ^3^ Department of Medicinal Chemistry, School of Pharmacy, Shiraz University of Medical Sciences, Shiraz, Iran

**Keywords:** anticancer agents, kinase inhibitors, multitargeted compounds, drug design, three-dimensional culture

## Abstract

The advent of novel receptor tyrosine kinase inhibitors has provided an important therapeutic tool for cancer patients. In this study, a series of quinazolinone hydrazide triazole derivatives were designed and synthesized as novel MET (c-MET) receptor tyrosine kinase inhibitors. The antiproliferative effect of the synthesized compounds was examined against EBC-1, A549, HT-29 and U-87MG cells by MTT assay. MET kinase inhibitory effect was tested by a Homogenous Time Resolved Fluorescence (HTRF) assay. The antiproliferative effect of compounds in a three-dimensional spheroid culture was studied by acid phosphatase (APH) assay, while apoptosis induction was examined by Hoechst 33258 staining. We found that compound **CM9** bearing p-bromo benzyl pendant inhibited MET kinase activity at the concentrations of 10–50 μM (% Inhibition = 37.1–66.3%). Compound **CM9** showed antiproliferative effect against cancer cells, in particular lung cancer cells with MET amplification (EBC-1) with an IC_50_ value of 8.6 μM. Moreover, this derivative inhibited cell growth in spheroid cultures in a dose-dependent manner and induced apoptosis in cancer cells. Assessment of inhibitory effect of **CM9** against a panel of 18 different protein kinases demonstrated that this compound also inhibits ALK, AXL, FGFR1, FLT1 (VEGFR1) and FLT4 (VEGFR3) more than 50% at 25 μM. Finally, molecular docking and dynamics simulation corroborated the experimental findings and showed critical structural features for the interactions between **CM9** and target kinases. The findings of this study present quinazolinone hydrazide triazole derivatives as kinase inhibitors with considerable anticancer effects.

## 1 Introduction

Cancer is the first or second leading cause of death before the age of 70 in 112 countries, causing 10 million deaths in 2020 worldwide (Sung et al., 2021). Various studies have focused on cancer treatment approaches targeting the signaling pathways in cancer cells and, in particular, protein kinases (Bhullar et al., 2018). It is well established that genetic aberrations in receptor tyrosine kinases (RTKs), such as MET (mesenchymal-epithelial transfer factor tyrosine or c-MET), VEGFR-1/2/3 (vascular endothelial growth factor receptor 1/2/3) (Qin et al., 2020), EGFR (epidermal growth factor receptor) (Ayati et al., 2020), RET (rearranged during transfection), FLT3 (FMS-like tyrosine kinase receptor 3) (Cilibrasi et al., 2022) and AXL (AXL receptor tyrosine kinase) (Okura et al., 2020), among others, contribute to tumorigenesis, disease aggressiveness, and drug resistance in different malignancies (Cohen et al., 2021) making RTKs promising therapeutic targets. Hence, substantial efforts have been devoted to the search for novel anticancer agents to pharmacologically modulate RTK signaling pathways, representing itself in the FDA approval of many inhibitors for clinical management of cancer patients (Zhao et al., 2021). MET receptor is an important oncogenic RTK that has received much attention as a promising drug target in various malignancies (Fogli et al., 2022). This receptor, also named as hepatocyte growth factor receptor (HGFR), is activated by binding to its natural ligand, HGF/scatter factor (Organ and Tsao, 2011). The aberrant activation of HGF/MET signaling pathway arises from overexpression, MET gene amplification or activating mutations, as well as excessive autocrine or paracrine HGF secretion, which has been reported to be associated with the development and progression of many types of cancers including lung, renal, gastrointestinal, thyroid, and breast cancers as well as glioblastoma among others (Zhang et al., 2018; Fu et al., 2021).

**FIGURE 1:**
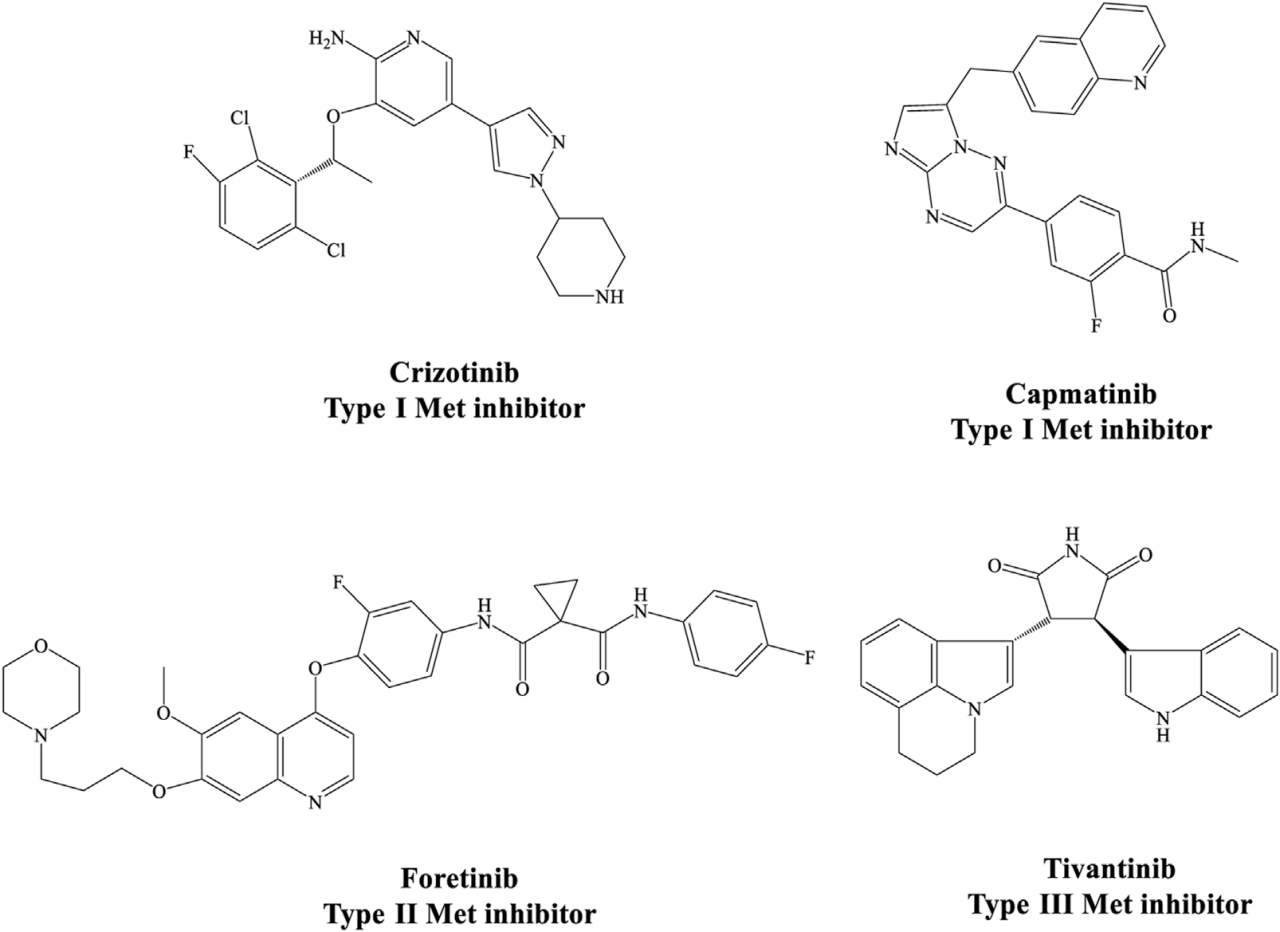
Different structural types of small-molecule MET kinase inhibitors.

Considering the important oncogenic role of this receptor, many small molecule kinase inhibitors are being studied with the aim of targeting HGF/MET signaling pathway in different tumors. These agents can be divided into ATP competitive (type I and II) and non-competitive (type III) inhibitors based on their binding modes and selectivity profiles (Figure 1) (Merchant et al., 2013; Zhang et al., 2015). More selective inhibitors of MET kinase include type I agents such as capmatinib with a U-shaped structure (Dhillon, 2020), while type II agents are high molecular weight compounds that occupy the hydrophobic back pocket of MET, leading to increased interactions with the hydrophobic site (Yuan et al., 2020). Multiple kinases are typically inhibited by type II MET inhibitors. Examples include foretinib, cabozantinib, and BMS777607, which have been approved by the FDA or are currently in clinical trials across a broad range of cancer types (Underiner et al., 2010; Yakes et al., 2011).

**FIGURE 2:**
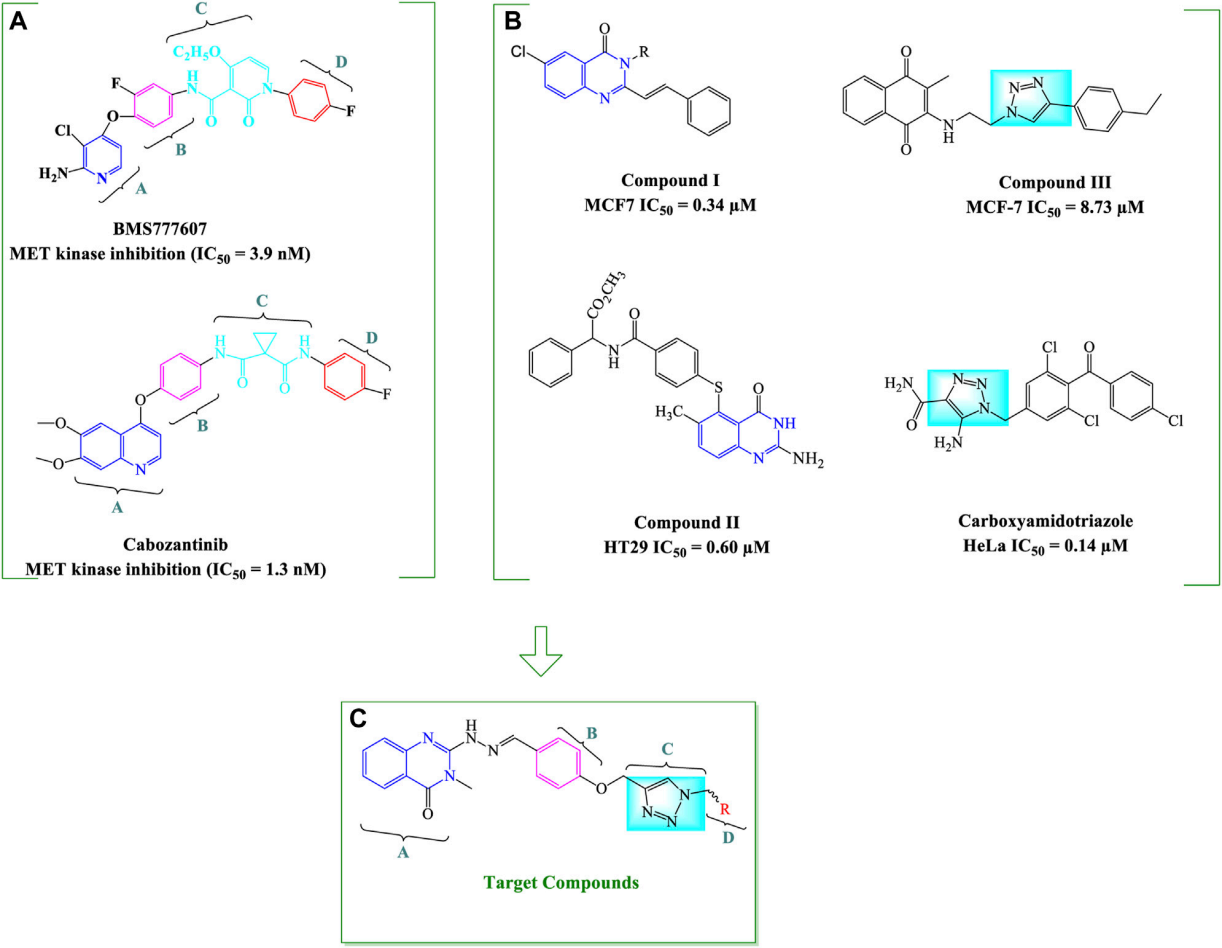
General strategy for the design of quinazolinone-hydrazide-triazole hybrids. The important structural features of two type II MET inhibitors BMS777607 and cabozantinib **(A)** and chemical structures of some potent antiproliferative compounds bearing 4-quinazolinone and 1,2,3-triazole moieties **(B)** as well as the general structure of designed quinazolinone-hydrazide-triazole hybrids **(C)** are shown (Baek et al., 2004; Raffa et al., 2004; Underiner et al., 2010; Yakes et al., 2011; Xu et al., 2019).

The general structural characteristics of Type II MET inhibitors consist of four distinct parts of A, B, C and D as shown in Figure 2A. Structure–activity relationship (SAR) studies of type II MET inhibitors have suggested that fragment A usually consists of nitrogen containing heterocyclic moieties such as quinolines, quinazolinone, and pyridines which have a great potential for hydrogen bonding interactions with the amino acid residues of the kinase domain (Li et al., 2013; Damghani et al., 2019). Moreover, quinazolinone derivatives such as compound I and II have been reported to have potent cytotoxic activities, and also, they were used as an important core in the structure of kinase inhibitors (Figure 2B) (Baek et al., 2004; Raffa et al., 2004; Mirgany et al., 2021). On the other hand, B and D fragments are usually a phenyl or substituted phenyl ring in the more promising compounds. The C fragment provides the five-atom linker with the capability of establishing H-bond interactions with the active site (Li et al., 2013; Tang et al., 2016). We and other investigators have previously employed 1,2,3-triazole as C linker for design of MET kinase inhibitors; which is a privileged heterocycle extensively used for the design of potent cytotoxic agents such as carboxyamidotriazole (Duan et al., 2013; Mareddy et al., 2017; Gholampour et al., 2019; Xu et al., 2019; Damghani et al., 2021) (Figure 2B).

We focused on the design of new antiproliferative agents with potential interactions with MET active site as type II inhibitors by applying the principles of molecular hybridization and bioisosteric replacement. Therefore, quinazolinone core was employed as part A, hydrazide moiety was selected as a linker between A and B fragments to enhance the hydrogen bonding interactions with the active site and the phenyl ring was applied as a central aryl ring (part B). The nitrogen atoms in 1,2,3-triazole and oxygen atoms in the linker were applied as potential H-bond acceptor or donor interacting moieties to provide favorable H-bond interactions with the kinase active site. Finally, different substituted benzyl derivatives and heteroaromatic pendants were utilized as part D in order to assess the structure-activity relationship of designed analogues (Figure 2C).

Upon the design and synthesis of anticancer agents with MET kinase inhibitory potential, the antiproliferative effect of compounds against cancer cell lines was evaluated in monolayer and three-dimensional (3D) cell cultures and the kinase inhibitory activity of synthesized derivatives was determined. *In silico* studies were also performed in order to investigate the binding interactions of the most promising compounds with the active site of target kinases.

## 2 Material and methods

### 2.1 Chemistry

Melting points were determined with a Thermo Scientific Electrothermal digital apparatus (Thermo Fisher Scientific Inc.). ^1^H NMR (300 MHz) and ^13^C NMR (100 MHz) spectra were recorded on a Bruker 300 Fourier transform spectrometer; the chemical shifts are expressed in d (ppm) downfield from tetramethylsilane. Infrared (IR) spectra were recorded on the Perkin Elmer Spectrum RXI FTIR spectrophotometer in the KBr phase. Mass spectra were carried out using Agilent 7000 triple quadrupole mass spectrometer at an electron impact mode with an ionization voltage of 70 eV.Chemicals used were supplied from Sigma-Aldrich, Fluka, and Merck chemical companies. TLC was performed on the glass-backed silica gel sheets (Silica Gel 60 GF254) and visualized under UV light (254 nm).

#### 2.1.1 Synthesis of 2-mercapto-3-methylquinazolin-4(3H)-one (Bhullar et al., 2018)

Anthranilic acid (**1**, 20 mmol) and triethylamine (2 ml) were dissolved in EtOH (10 ml) and methyl isothiocyanate (30 mmol) was added to the mixture. The reaction mixture was heated under reflux for 2 h. After cooling, the precipitate was filtered and washed 3 times with cold ethanol, dried and recrystallized from ethanol (Haghighijoo et al., 2017).

#### 2.1.2 Synthesis of 2-hydrazinyl-3-methylquinazolin-4(3H)-one (Qin et al., 2020)

A mixture of compound 2 (1 mmol) and hydrazine hydrate (1 ml, 32 mmol) in BuOH (20 ml) was refluxed for 20 h. After cooling, the precipitated solid was separated and recrystallized from diethyl ether to give pure compound 3 (Haghighijoo et al., 2017).

#### 2.1.3 Synthesis of 4-(prop-2-yn-1-yloxy)benzaldehyde (Cilibrasi et al., 2022)

4-hydroxybenzaldehyde (**4**, 1.22 g, 10 mmol) and K_2_CO_3_ (2.57 g, 15 mmol) were dissolved in 100 ml dry acetone and stirred for 20 min. Then, propargyl bromide (3.57 g, 30 mmol) was added and the reaction mixture was heated under reflux for 6 h. After cooling, the precipitate was filtered and the solvent was evaporated. Finally, the residue was recrystallized from ethanol (Yazdani et al., 2019; Damghani et al., 2021).

#### 2.1.4 Synthesis of different benzyl azides (Cohen et al., 2021)

Appropriate benzyl halide (**6**, 1.1 mmol) and sodium azide (0.0585g, 0.9 mmol) were dissolved in a mixture of H_2_O/isopropanol (5 ml, 1:1) and the reaction mixture was stirred at room temperature for 30–45 min. After completion of the reaction as indicated by TLC, the reaction mixture would be ready for the next step.

#### 2.1.5 Synthesis of different 4-((1-benzyl-1H-1,2,3-triazol-4-yl)methoxy)benzaldehyde (Zhao et al., 2021)

4-(Prop-2-ynyloxy) benzaldehyde (**5**, 0.08 g, 0.5 mmol), CuSO_4_ (5 mol %), and sodium ascorbate (15 mol %) were added to the reaction mixture in previous step and were stirred at room temperature for 48 h. In the next step, the mixture was concentrated under reduced pressure and diluted with ethyl acetate and washed with water. The organic extracts were dried over anhydrous sodium sulfate and concentrated under reduced pressure. This intermediate have been previously reported by Kumar et al. (2013).

#### 2.1.6 General procedure for the synthesis 2-(2-(4-((1-benzyl-1H-1,2,3-triazol-4yl)methoxy) benzylidene)hydrazineyl)-3-methylquinazolin-4(3H)-one derivatives (CM1-CM10)

To a solution of different 4-((1-benzyl-1H-1,2,3-triazol-4-yl)methoxy)benzaldehyde (**8**, 1 mmol) in absolute ethanol (5 ml) was added few drops of acetic acid and stirred at room temperature. Then, 2-hydrazinyl-3-methylquinazolin-4(3H)-one (**3**, 0.19 g, 1 mmol) dissolved in absolute ethanol (5 ml) was added to the stirred reaction solution and the mixture was refluxed overnight. After completion of the reaction as indicated by TLC, the reaction mixture was allowed to cool in room temperature. The precipitated product was filtered and recrystallized from acetone.

(E)-3-methyl-2-(2-(4-((1-(4-nitrobenzyl)-1H-1,2,3-triazol-4-yl)methoxy)benzylidene) hydrazineyl)quinazolin-4(3H)-one (CM1). Yellow solid; Yield 88%; mp: 201–203°C; ^1^H NMR (300 MHz, DMSO- d6) δ (ppm): 10.18 (br s, 1H, NH), 8.38 (s, 1H, N=CH), 7.09-8.29 (m, 13H, quinazolinone-H, Ar-H, triazole-H), 5.79 (s, 2H, CH_2_-O), 5.28 (s, 2H, CH_2_-N), 2.46 (s, 3H, N-CH_3_); ^13^C NMR (100 MHz, DMSO *d6*) δ (ppm): 175.36, 159.91(2C), 153.04, 143.35, 139.51, 134.57, 129.97, 129.13, 129.06, 129.01, 128.94, 128.73, 127.55, 124.37, 123.86, 121.67, 114.86, 114.16, 61.57, 52.43, 27.22; IR (KBr) (ʎ_max_/cm^1^): 3344 (NH), 1682 (C=N), 1616 (C=O), 1240 (C-N), 1007 (C-O).

(E)-3-methyl-2-(2-(4-((1-(4-methylbenzyl)-1H-1,2,3-triazol-4 yl)methoxy)benzylidene) hydrazineyl)quinazolin-4(3H)-one (CM2). Bright yellow solid; Yield 55%; mp: 146–148°C; ^1^H NMR (300 MHz, DMSO d6) δ (ppm): 9.07 (br s, 1H, NH), 8.35 (s, 1H, N=CH), 6.83-8.24 (m, 13H, quinazolinone-H, Ar-H, triazole-H), 5.48 (s, 2H, CH_2_-O), 5.15 (s, 2H, CH_2_-N), 3.4 (s, 3H, N-CH3), 2.2 (s, 3H, CH3); ^13^C NMR (100 MHz, DMSO d6) δ (ppm): 170.81, 153.81, 153.36, 134.86, 134.76 (2C), 129.85, 129.20, 129.06, 128.90, 128.36, 128.23(2C), 128.17, 122.78, 122.42. 114.97, 114.54, 114.22, 61.14, 52.76, 34.78, 28.0; IR (KBr) (ʎ_max_/cm^1^): 3355 (NH), 1682 (C=N), 1616 (C=O), 1243 (C-N), 1005 (C-O).

**(**E)-2-(2-(4-((1-(4-(tert-butyl)benzyl)-1H-1,2,3-triazol-4-yl)methoxy)benzylidene)hydrazineyl)-3-methylquinazolin-4(3H)-one (CM3). White solid; Yield 60%; mp: 173–175°C; ^1^H NMR (300 MHz, DMSO d6) δ (ppm): 8.42 (m, 2H, NH, N=CH), 7.09-8.29 (m, 13H, quinazolinone-H, Ar-H, triazole-H), 5.53 (s, 2H, CH_2_-O), 5.32 (s, 2H, CH_2_-N), 3.63 (s, 3H, N-CH3), 1.29 (s, 9H, t-Bu); ^13^C NMR (100 MHz, DMSO d6) δ (ppm): 174.66, 159.58, 156.01, 155.63, 152.66, 149.07, 148.11, 145.92, 143.76, 141.55, 139.69, 138.74, 134.93, 129.53, 128.15, 127.52, 126.53, 123.77, 121.37, 67.89, 61.08, 34.10, 33.61, 28.77; IR (KBr) (ʎ_max_/cm^1^): 3364 (NH), 1681 (C=N), 1618 (C=O), 1244 (C-N), 1005 (C-O).

(E)-2-(2-(4-((1-(4-fluorobenzyl)-1H-1,2,3-triazol-4-yl)methoxy)benzylidene)hydrazineyl)-3-methylquinazolin-4(3H)-one (CM4). Green solid; Yield 80%; mp: 162–164°C; ^1^H NMR (300 MHz, DMSO d6) δ (ppm): 9.22 (br s, 1H, NH), 8.43 (s, 1H, N=CH), 6.93-8.12 (m,, 13H, quinazolinone-H, Ar-H, triazole-H), 5.54 (s, 2H, CH_2_-O), 5.56 (s, 2H, CH_2_-N), 3.54 (s, 3H, N-CH3); ^13^C NMR (100 MHz, DMSO d6) δ (ppm): 153.77, 150.32, 144.34, 138.34, 135.45, 134.86, 130.25, 130.19, 130.12, 130.01, 129.90, 129.20, 128.44, 128.38, 125.10, 122.65, 116.37, 116.08, 114.95, 114.28, 112.92, 62.06, 53.57, 29.71; IR (KBr) (ʎ_max_/cm^1^): 3364 (NH), 1683 (C=N), 1616 (C=O), 1241 (C-N), 1006 (C-O).

(E)-2-(2-(4-((1-(4-chlorobenzyl)-1H-1,2,3-triazol-4-yl)methoxy)benzylidene)hydrazineyl)-3-methylquinazolin-4(3H)-one (CM5). Yellow solid; Yield 85%; mp: 191–193°C; 1H NMR (300 MHz, DMSO d6) δ (ppm): 9.14 (br s, 1H, NH), 8.33 (s, 1H, N=CH), 6.91-7.63 (m, 13H, quinazolinone-H, Ar-H, triazole-H), 5.44 (s, 2H, CH2-O), 5.15 (s, 2H, CH2-N), 3.54 (s, 3H, N-CH3); 13C NMR (100 MHz, DMSO d6) δ (ppm): 161.16, 159.72, 153.69, 150.37, 138.44, 134.92, 134.79, 132.89, 132.00, 131.87, 130.16, 129.46, 129.38, 129.13, 128.29, 122.75, 122.29, 114.93, 114.12, 62.01, 53.53, 27.93; IR (KBr) (ʎmax/cm1): 3342 (NH), 1667 (C=N), 1622 (C=O), 1246 (C-N), 1004 (C-O).

(E)-2-(2-(4-((1-benzyl-1H-1,2,3-triazol-4-yl)methoxy)benzylidene)hydrazineyl)-3-methylquinazolin-4(3H)-one (CM6). White solid; Yield 65%; mp: 176–178°C; ^1^H NMR (300 MHz, DMSO *d6*) δ (ppm): 9.09 (br s, 1H, NH), 8.41 (s, 1H, N=CH), 6.58-8.13 (m, 14H, quinazolinone-H, Ar-H, triazole-H), 5.56 (s, 2H, CH_2_-O), 5.24 (s, 2H, CH_2_-N), 3.47 (s, 3H, N-CH3); ^13^C NMR (100 MHz, DMSO *d6*) δ (ppm): 161.22, 161.04, 159.78, 153.77, 153.31, 150.40, 138.44, 134.80, 133.68, 129.20, 129.12, 128.98, 128.90, 128.46, 128.40, 128.16, 124.62, 122.24, 114.97, 61.13, 52.75, 27.90; IR (KBr) (ʎ_max_/cm^1^): 3365 (NH), 1667 (C=N), 1620 (C=O), 1234 (C-N), 1005 (C-O).

(E)-2-(2-(4-((4-((2-(3-methyl-4-oxo-3,4-dihydroquinazolin-2-yl)hydrazineylidene) methyl) phenoxy)methyl)-1H-1,2,3-triazol-1-yl)ethyl)isoindoline-1,3-dione (CM7). White solid; Yield 90%; mp: 170–172°C; ^1^H NMR (300 MHz, DMSO d6) δ (ppm): 9.22 (br s, 1H, NH), 8.13 (s, 1H, N=CH), 7.02-7.67 (m, 13H, quinazolinone-H, phenyl-H, triazole-H, isoindoline-1,3-dione-H), 4.76 (s, 2H, CH_2_-O), 4.13 (s, 2H, CH_2_-N), 3.54 (s, 3H, N-CH3); ^13^C NMR (100 MHz, DMSO d6) δ (ppm): 190.07, 176.54, 169.91, 160.55, 159.05, 152.95, 150.31, 139.50, 139.46, 139.39, 139.39, 138.85, 134.56, 129.05, 127.55, 121.68, 115.14, 114.89, 114.16, 76.37, 55.48, 27.23; IR (KBr) (ʎ_max_/cm^1^): 3400 (NH), 1668 (C=N), 1609, 1590 (C=O), 1248 (C-N), 1012 (C-O).

(E)-2-(2-(4-((1-(3,4-dichlorobenzyl)-1H-1,2,3-triazol-4-yl)methoxy)benzylidene)hydrazinyl)-3-methylquinazolin-4(3H)-one (CM8). Green solid; Yield 90%; mp: 142–144°C; 1H NMR (300 MHz, DMSO d6) δ (ppm): 8.32 (s, 1H, N=CH), 7.03-799 (m, 13H, quinazolinone-H, phenyl-H, triazole-H, N-H), 5.60 (s, 2H, CH2-O), 5.16 (s, 2H, N -CH2), 3.32 (s, 3H, N-CH3; 13C NMR (100 MHz, DMSO d6) δ (ppm): 177.96, 163.96, 160.72, 159.85, 143.20, 142.64, 132.71, 132.67, 130.88, 130.76, 129.37, 127.78, 127.49, 125.21, 122.49, 119.51, 118.19 (2C), 116.22, 115.94, 115.34, 61.56, 52.49, 27.11; IR (KBr) (ʎmax/cm1): 3400 (NH), 1668 (C=N), 1621 (C=O), 1243 (C-N), 1028 (C-O).

(E)-2-(2-(4-((1-(4-bromobenzyl)-1H-1,2,3-triazol-4-yl)methoxy)benzylidene)hydrazinyl)-3-methylquinazolin-4(3H)-one (CM9). White solid; Yield 75%; mp: 187–189°C; 1H NMR (300 MHz, DMSO d6) δ (ppm): 7.99 (s, 1H, N=CH), 7.95 (br s, 1H, NH), 7.02-7.67 (m, 13H, quinazolinone-H, Ar-H, triazole-H), 5.63 (s, 2H, CH2-O5.18 (s, 2H, CH2-N), 3.41 (s, 3H, N-CH3); 13C NMR (100 MHz, DMSO d6) δ (ppm): 177.96, 172.61, 159.61, 159.83, 144.92, 143.29, 142.66, 138.78, 137.02, 133.75, 131.21, 129.37, 128.65, 128.30, 128.07, 127.50, 127.17, 126.85, 125.43, 122.55, 115.36, 114.97, 61.54, 52.52, 30.87; IR (KBr) (ʎmax/cm1): 3400 (NH), 1668 (C=N), 1609, 1590 (C=O), 1248 (C-N), 1012 (C-O).

(E)-2-(2-(4-((4-((2-(3-methyl-4-oxo-3,4-dihydroquinazolin-2-yl)hydrazono)methyl)phenoxy) methyl)-1H-1,2,3-triazol-1-yl)ethyl)isoindoline-1,3-dione (CM10). White solid; Yield 83%; mp: 161–163°C; ^1^H NMR (300 MHz, DMSO d6) δ (ppm): 11.36 (br s, 1H, NH), 7.00-8.27 (m, 14H, quinazolinone-H, Ar-H, triazole-H, N=CH), 4.85 (s, 2H, CH_2_-O), 3.64 (m, 4H, CH_2_-N), 3.54 (s, 3H, N-CH3); 2.14 (t, 2H, CH_2_); ^13^C NMR (100 MHz, DMSO d6) δ (ppm): 178.01, 172.51, 168.56, 163.55, 160.20, 158.98, 154.31, 152.16, 142.48, 134.81, 132.20, 130.56, 129.36, 127.88, 125.28, 123.45, 120.23, 119.61, 115.46, 78.96, 55.96, 36.64, 31.56, 21.54

### 2.2 Cell culture

EBC-1 (human lung adenocarcinoma cells) were obtained from Japanese Collection of Research Bio Resources Cell Bank (JCRB). A549 (human lung adenocarcinoma cells), HT-29 (Human colorectal adenocarcinoma cells) and U-87MG (human primary glioblastoma cells) were obtained from Iranian Biological Resource Centre, Tehran, Iran. EBC-1 cells were cultured in RPMI 1640 medium containing 10% heat-inactivated fetal bovine serum (FBS) and 1% penicillin/streptomycin. A549, HT-29 and U-87MG cells were grown in DMEM low glucose, containing 10% heat-inactivated FBS and 1% penicillin/streptomycin. All cells were grown in monolayer culture at 37°C in a humidified incubator with 5% CO_2_.

### 2.3 Assessment of the antiproliferative effect by MTT assay

MTT (3-(4,5-dimethylthiazol-2-yl)-2,5-diphenyltetrazolium bromide) assay was applied to evaluate the antiproliferative effects of synthetic compounds against cancer and normal cell lines as previously described (Damghani et al., 2021). The MTT assay is based on the reduction function of NAD(P)H-dependent cellular oxidoreductase enzymes on yellow tetrazolium dye MTT to its insoluble formazan, which has a purple color and reflects the number of viable cells. Cancer and non-cancer cells were seeded in 96-well flat bottom plates at different densities of 1.5 × 10^4^ (HT-29), 3 × 10^4^ (A549 and U-87MG), 4 × 10^4^ (EBC-1). Synthetic compounds were first dissolved in DMSO and then diluted in growth medium. After 24 h the attached cells were exposed to 100 µL of 3-4 different concentrations of synthesized derivatives in triplicate manner and incubated for an additional 72 h at 37°C. In addition, 3–4 different concentrations of reference compounds were added to wells. To perform the MTT assay the media was discarded from each well and MTT solution in phosphate-buffered saline at a concentration of 0.5 mg/ml was added to all wells. Then, plates were incubated at 37°C for 4 h to allow formation of formazan crystals. Afterwards, crystals were dissolved in 200 μL DMSO, shake on an orbital shaker for 30 min and absorbance was measured at a wavelength of 570 nm with background correction at 650 nm using a Bio-Tek microplate reader (Model Synergy HTX). Finally, IC_50_ values for each compound was calculated with CurveExpert software version 1.4 for windows. Each experiment was repeated 3–5 times.

### 2.4 *In vitro* enzymatic assays

MET kinase inhibitory activity of synthesized derivatives was determined by Homogeneous Time Resolved Fluorescence (HTRF) assay (Damghani et al., 2021). In this method, the phosphorylation level of a biotinylated tyrosine kinase substrate peptide (TK substrate) is measured. The HTRF^®^ KinEASE™ TK kit and MET kinase were purchased from Cisbio and Millipore, respectively. The optimal conditions were established for enzyme, substrate, ATP concentrations, and enzymatic reaction time. Test compounds were first dissolved in DMSO and then diluted in the reaction buffer containing 50 mM HEPES pH 7.0, 0.1 mM sodium orthovanadate, 0.01% BSA, 0.02% NaN_3_, 10 mM MgCl_2_, 5 mM MnCl_2_, 2 mM DTT. Briefly, 4 μL of test compound at different concentrations (10, 25, 50 and 100 μM final concentrations) were loaded in a white 384-well plate (Cisbio Cat Number: 6007299). Then, 2 μL of MET kinase diluted in kinase buffer (0.25 ng/μL) were preincubated with 4 μL of the test derivatives for 10 min. In the next step, in order to initiate the reaction, 2 μL of TK substrate (1 μM final concentration), and 2 μL ATP dissolved in kinase buffer (25 μM final concentration) were sequentially added. After the incubation of the reaction mixture for 50 min at room temperature, the phosphorylated peptide substrate was detected by adding 10 μL mixed detection solution containing 5 μL Europium^3+^-conjugated antibody and 5 μL Steptavidin-XL665 (125 nM final concentration). Finally, the Time Resolved-Fluorescence Resonance Energy Transfer (TR-FRET) signal was determined after an hour of incubation at room temperature. The plate was read at excitation of 337 nm and dual emissions of 665 and 620 nm using a Bio-Tek multimode plate reader (Model Cytation 3). The following equations were used to determine the inhibition rate (%):

$${Ratio}_{665/620}={Emission}_{665\;nm}\;/\;{Emission}_{620\;nm}$$

$$\Delta R=\backslash \left(Ratio\;{Sample}_{665/620}-Ratio\;{Background}_{665/620}\right)*100/\;Ratio\;{Background}_{665/620}$$

$$Inhibition\;(\%)={(}_{\Delta RControl}-\Delta R_{Sample})*100\;/\;\Delta R_{Control}$$

Background correction samples contained all reagents except for the enzyme. Control wells contained the same amount of DMSO contained in the sample. The final concentration of DMSO did not exceed 2%.

Kinase inhibition profile was examined by Kinase Radiometric Assays for 18 different oncogenic kinases (Eurofins Cerep, Celle l'Evescault, France).

### 2.5 Measurement of the anticancer effect in three-dimensional spheroid assay

An agarose-based liquid overlay method was implemented to perform three-dimensional spheroid cell cultures assays. To this purpose, agarose solution in RPMI (1.5% w/v) was prepared and high-pressure sterilized. The wells of a 96-well sterile flat bottom plate were coated with 50 µL sterilized agarose solution and allowed to become gelatinous at room temperature for at least 2 h. A suspension of EBC-1 cells in RPMI medium containing 10% FBS at a density of 2 × 10^5^ cells/ml were added to each well (125 µL per well). Then, In order to allow the formation of a single spheroid in each well, the plate was centrifuged at 700 g for 5 min and incubated under standard culture conditions for 48 h. Afterwards, formed spheroids were exposed to 3-4 different concentrations of active derivatives diluted in a fresh medium containing 10% FBS. After 72 h incubation period, the images of spheroids were recorded by a bright field microscope (Nikon model DS-Ri2) and Nikon NIS-Elements AR imaging software for Windows version 4.30. The obtained images were analysed with ImageJ software for windows. In addition, cell viability was measured by a colorimetric acid phosphatase (APH) assay. The APH assay used p-nitrophenyl phosphate (pNPP) as substrate which dephosphorylates and converts to yellow p-nitrophenol by intracellular acid phosphatases present in viable cells. Briefly, 160 µL of the medium was disposed and 200 µL of APH solution containing 2 mg/ml pNPP dissolved in 0.1 M sodium acetate at pH 4.8 were added to each well and incubated for 120 min. Afterwards, 10 µL of 1 M NaOH was added to each well as the stop solution. Finally, the absorbance was recorded at 405 nm within 10 min by a Bio-Tek microplate reader (Model Synergy HTX).

### 2.6 Assessment of apoptosis induction by Hoechst 33258 staining

In order to evaluate the apoptosis induction effects of test compounds on cancer cells, Hoechst 33258 staining was applied. Hoechst dye is a member of blue fluorescent nuclear specific dyes which binds to DNA and stains nuclei in live and fixed cells. EBC-1 cells were cultured in 6-well plates with a density of 5 × 104 cells/ml (2 ml per well). After 24 h incubation, the whole culture medium from all wells was removed and 2-3 different concentrations of active derivatives were added to each well and incubated for 72 h. Then, the whole culture medium was replaced with 1 ml of 4% cold freshly prepared paraformaldehyde (PFA) and cells were incubated for 20 min at room temperature allow cells were fixed. Afterward, the cells were washed two times with PBS and incubated with 1 ml Hoechst 33258 2.5 μg/ml for 30 min at room temperature in the dark place. Finally, the cells were washed with PBS and imaged with a fluorescence microscope (Nikon model DS-Ri2).

### 2.7 *In silico* studies

#### 2.7.1 Homology modeling

The crystal structure of VEGFR2 (PDB code: 1YWN) was used as a template for human FLT4 (VEGFR3) homology modeling; the sequence identity between VEGFR2 and FLT4 (VEGFR3) is more than 75%. The homology model of FLT4 (VEGFR3) was generated by the I-TASSER modeling server. The constructed model was subjected to the MD simulation for 100 ns. The established FLT4 (VEGFR3) homology model was then applied for the docking study. The key residues in the binding site of FLT4 (VEGFR3) include Cys930, Ala877, Val859, Phe929, Leu1044, Lys879, Asp1055, Cys1054, Val910, Glu896, Ile899, Leu900, Leu851, Asn934, Arg1041, Asn 937, and Arg984 (Xu et al., 2015; Li et al., 2021).

#### 2.7.2 Docking analysis

Molecular docking analysis was carried out against MET and FLT4 (VEGFR3) to investigate the binding modes and the critical molecular interactions between the synthetic compounds and the binding site of the targets, using the smina molecular docking software (Koes et al., 2013). smina was developed based on Auto-Dock Vina to enhance scoring function development and energy minimization. The protein structure was prepared using adding hydrogens removing water molecules and native ligands. Then, the Kollmann charges were assigned to the receptor. All compounds were sketched using the Marvin Sketch (http://www.chemaxon.com), and assigned gasteiger charges and energy optimization of synthetic compounds using the steepest descent algorithm carried out by Open Babel (O'Boyle et al., 2011). The enzyme’s binding site for the docking process was determined automatically using the coordinates of co-crystallized native ligand foretinib with MET kinase. Then, smina was applied to predict the interaction and binding modes of the ligands inside the enzyme active site. The computational docking approach was evaluated based on the root-mean-square deviation (RMSD) value from re-docking of the co-crystalized ligand foretinib back into the active pocket site of the receptor (Gohlke et al., 2000).

#### 2.7.3 Molecular dynamics simulation

The molecular dynamics simulation (MD) was carried out using GROMACS package version 2019.1 on a GPU Linux server to simulate the interaction of MET and FLT4 (VEGFR3) with **CM9**. The Amber99sb force field was chosen to carry out the MD simulations at a mean temperature of 300 K and physiological pH 7. Chimera software (Pettersen et al., 2004) was applied to adding the AM1 partial charges to prepare the topology, and the algorithm acpype (Da Silva and Vranken, 2012) was used for the creation of force filed parameters (Da Silva and Vranken, 2012). A dodecahedral solute box was defined around the solute such that there is the shortest distance between any two periodic images of a protein complex and the edge of the box. TIP3P water types were used to fill the protein complex box. Then, 0.15 mol/lit sodium chloride was added to the system for neutralization by replacing the equal number of water molecules. System energy optimization was carried out using the steepest descent algorithm through a 100 ps run. In the next step, the macromolecule and ligand’s atom positions were restrained using a force constant of 1000 kJ mol^−1^ nm^−2^ during a 500 ps NVTperiod. During the NVT stage, the temperature was set to 300 K using V-rescale thermostats. Then, the pressure of the system was stabilized over the period of 500 ps equilibration step during the NPT step.

Generally, MD simulations need to be adequately long to draw logistic conclusions from the studies. The production MD simulation was completed during 100 ns for both complexes under a well-equilibrated system with a desired temperature and pressure. The long-ranged electrostatic contributions were calculated by the particle-mesh Ewald (PME) algorithm, and lengths of covalent bonds were restrained by applying the LINCS constraint algorithm, three to four times faster than the SHAKE algorithm. Upon terminating the MD run, the complex was centered by returning the protein to the box center, and the trajectory was corrected in the point of periodic boundary condition. The RMSD values were determined over the entire run for the alignment of the protein backbone atoms of each snapshot against the first frame as the reference to determine the equilibrium time range further. Moreover, cluster analysis of the trajectory during the equilibrium time range was applied by gromos method.

#### 2.7.4 Binding free energy calculations using MM-PBSA

The binding free energy of complexes of potent compound **CM9** with MET and FLT4 (VEGFR3) was determined using MM-PBSA approach. MM-PBSA analyses were performed using the g_mmpbsa tool provided by Kumari et al. (Baker et al., 2001; Kumari et al., 2014). MM-PBSA estimates the binding free energies using the combination of molecular mechanics and continuum solvent models. Besides the calculation of binding energy components, it can also report the individual energy contributions of amino acids. In this study, the last 1 ns of the equilibrium time range elucidated by the RMSD graph of all complexes was applied for precise MM-PBSA estimation. The adaptive Poisson-Boltzmann Solver (APBS) approach calculated the electrostatic energy, VDW energy, and polar solvation energy contributions. In contrast, the non-polar contributions of solvation energy were estimated by the Solvent-accessible surface area (SASA) approach. The grid spacing of 0.5 Å and probe radius of 1.4 Å were used for SASA estimate with a solvent dielectric constant value of 80, and solute dielectric constant value of 2. The binding energy of the complexes and energy contribution of ligand were specified at the end.

## 3 Results

### 3.1 Chemistry

**SCHEME 1:**
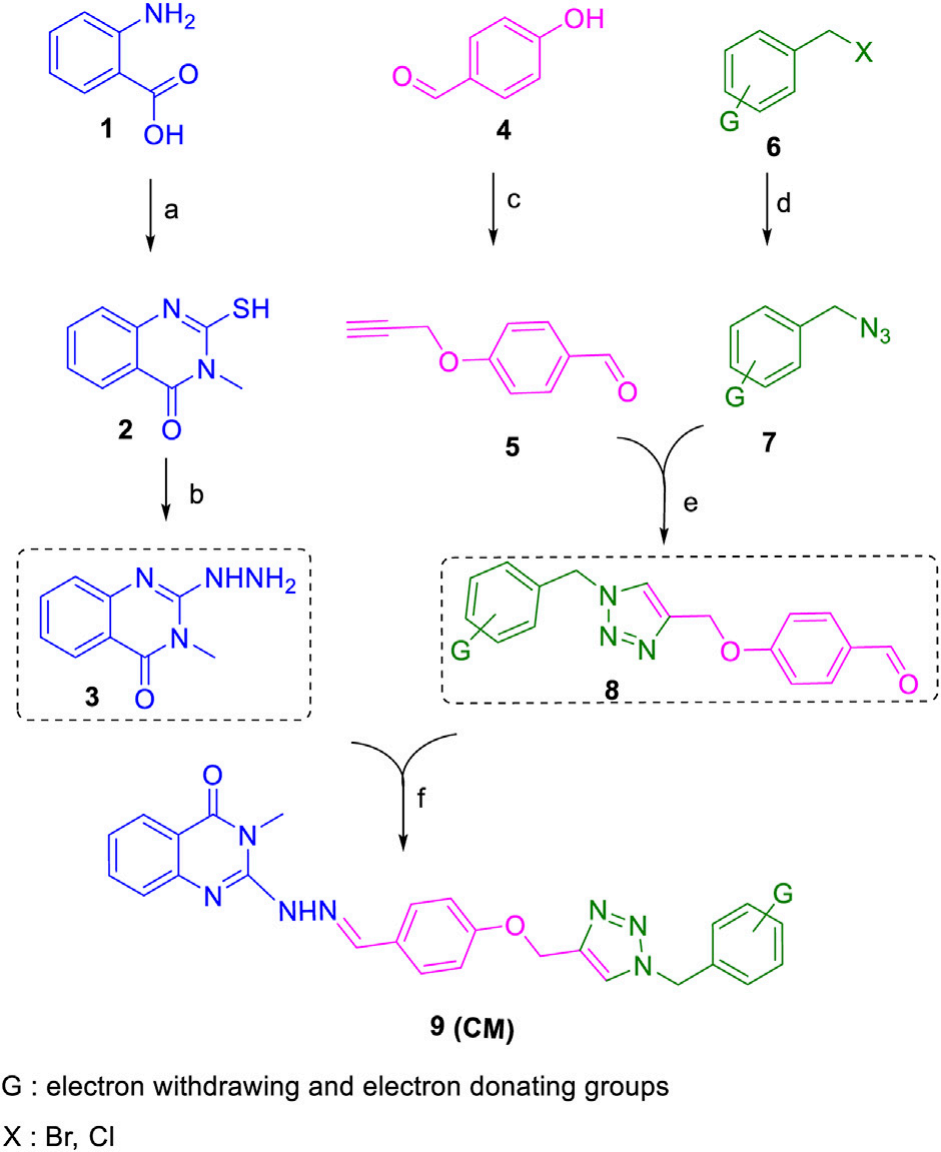
Copper‐catalyzed synthesis of new quinazolinone hydrazine derivatives (CM1-CM10). Reagents and conditions; a: Methyl isothiocyanate, Et_3_N, EtOH/reflux; b: N_2_H_4_.H_2_O, BuOH/reflux; c: propargyl bromide, K_2_CO_3_, dry acetone/reflux; d: different benzyl bromide or benzyl chloride, NaN_3_, Et_3_N, isopropanol, H_2_O/r.t. e: CuSO_4_, sodium ascorbate, isopropanol, H_2_O/r.t. f: AcOH (few drops), EtOH/reflux.

All target compounds **CM1-CM10** were synthesized according to the procedure shown in Scheme 1. At first, 2-mercapto-3-methyl quinazoline-4(3*H*)-one (Bhullar et al., 2018) was prepared *via* the reaction of anthranilic acid **1** and methyl isothiocyanate in the presence of triethylamine in refluxing ethanol. In the next step, the reaction of compound **2** with hydrazine hydrate under reflux condition in BuOH afforded 2-hydrazinyl-3-methyl quinazoline-4(3H)-one (Qin et al., 2020)**.** Then, 4-hydroxy aldehyde was set to react with 3-bromoprop-1-yne in the presence of K_2_CO_3_ in dry acetone/reflux to produce 4-(prop-2-yn-1-yloxy) benzaldehyde (Cilibrasi et al., 2022). Furthermore, different benzyl azides (Cohen et al., 2021) were prepared from the reaction of desired benzyl halide and sodium azide in a mixture of H_2_O/isopropanol at room temperature. The intermediate 4-((1-benzyl-1H-1,2,3-triazol-4-yl)methoxy)benzaldehyde (Zhao et al., 2021) was prepared *via* the reaction of compounds **5** and **7** in the presences of CuSO_4_ and sodium ascorbate in a mixture of H_2_O/isopropanol at room temperature. Finally, different derivatives (**CM1-CM10**) were synthesized through the reaction of compounds **8** and **3** in the presence of acetic acid in the absolute ethanol at room temperature (Scheme 1). All structures, characteristic chemical and physical properties of the compounds are demonstrated in Table 1.

**TABLE 1:** Chemical and physical properties of synthesized compounds **CM1**–**CM10**.

Compound	Chemical structure	m.p. (°C)	Yield (%)	Logp
CM1	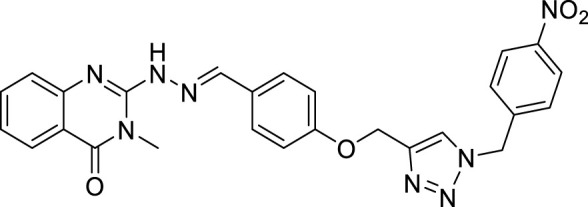	201–203	88	3.70
CM2	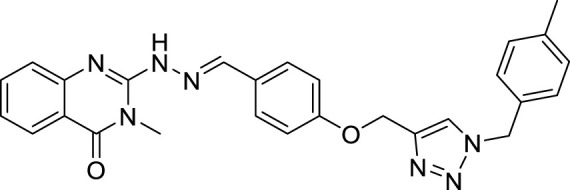	146–148	55	5.50
CM3	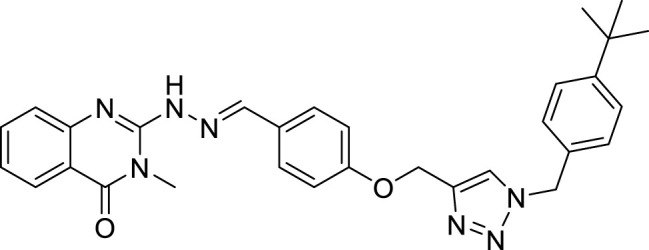	173–175	60	6.72
CM4	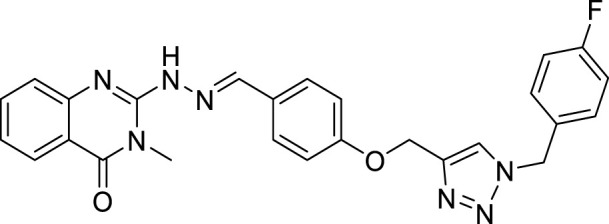	162–164	80	5.17
CM5	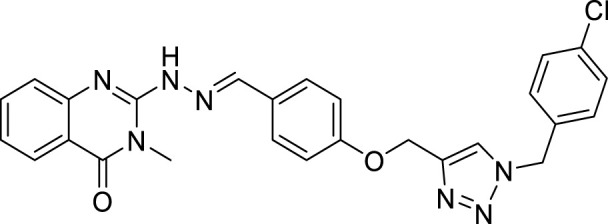	191–193	85	5.57
CM6	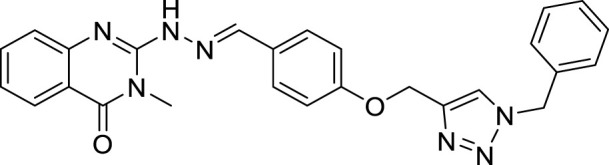	176–178	65	5.01
CM7	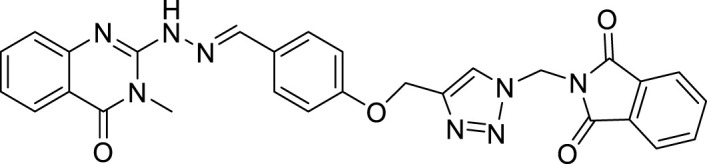	170–172	90	4.02
CM8	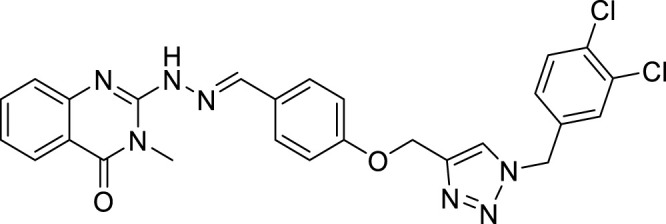	142–144	90	6.13
CM9	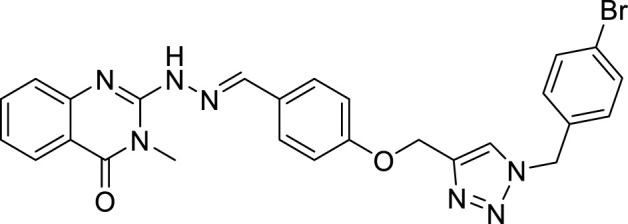	187–189	75	5.84
CM10	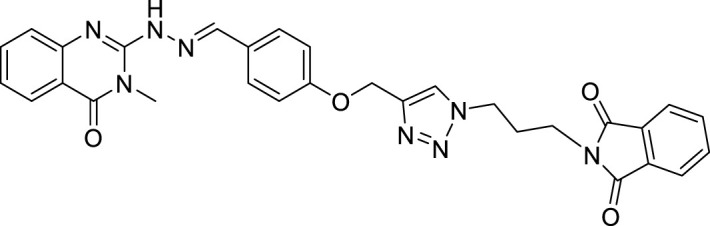	161–163	83	4.30

### 3.2 Antiproliferative effect against cancer cells

We employed the MTT assay in order to investigate the antiproliferative effects of the synthetic compounds against four different cancer cell lines, including lung cancer cell lines EBC-1 (with MET amplification) and A549, the human colorectal cancer cells HT-29, as well as the human glioblastoma cell line U-87MG. **CM7**, **CM8**, **CM9** and **CM10** were the most effective compounds at inhibiting the growth of the tested cancer cell lines, particularly EBC-1 cells with IC_50_ values ranging from 8.6 ± 1.9 to 22.9 ± 4.6 µM. Compound **CM9** (bearing para-bromophenyl moiety) displayed the highest growth inhibitory effect against the EBC-1 cell line harboring amplified *MET* gene. In addition to being effective against EBC-1 cells, the **CM9** and **CM10** derivatives exhibited good growth inhibitory effects against U-87MG and HT-29 cells with IC_50_ values of 18.4 ± 2.3 and 24.6 ± 2.6 µM, respectively (Table 2).

**TABLE 2:** Antiproliferative activity of synthetic quinazoline derivatives bearing various phenoxy-methylene-1,2,3-triazole pendants assessed by the MTT assay.

Compound	IC_50_ (µM)			
	Mean ± SEM			
	EBC-1	A549	HT29	U-87MG
CM1	>100	>100	>100	>100
CM2	>100	>100	>100	>100
CM3	>100	>100	56.4 ± 14.5	>100
CM4	36.2 ± 10.8	>100	27.8 ± 5.1	67.1 ± 6.2
CM5	>100	>100	>100	>100
CM6	32.5 ± 6.8	>100	>100	>100
CM7	22.9 ± 4.6	74.3 ± 9.5	>100	ND
CM8	22.4 ± 6.0	34.5 ± 2.2	47.8 ± 6.7	51.1 ± 7.6
CM9	8.6 ± 1.9	64.9 ± 2.9	65.2 ± 15.4	24.6 ± 2.6
CM10	18.0 ± 5.6	53.7 ± 2.7	18.4 ± 2.3	40.1 ± 1.7
Cabozantinib	59.2 ± 2.0 (nM)	16.2 ± 5.3	3.7 ± 0.4	2.3 ± 0.3

### 3.3 MET kinase inhibitory effect

**FIGURE 3:**
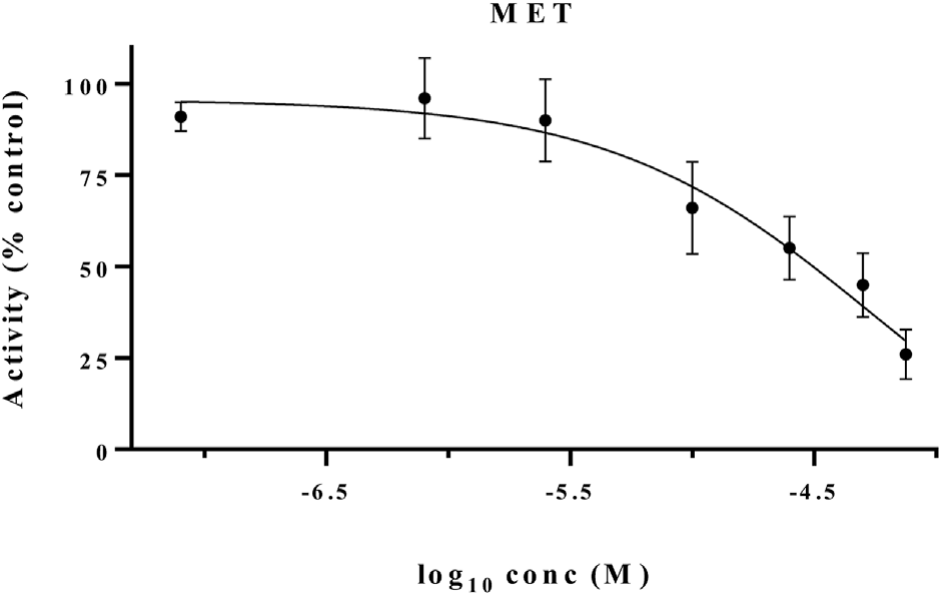
MET kinase inhibitory activity of **CM9**. A Homogeneous Time Resolved Fluorescence (HTRF) assay was performed as a cell free method for evaluation of MET kinase inhibitory activity of **CM9** compound. **CM9** inhibited MET kinase activity with an IC_50_ value of 22.76 µM.

The MET inhibitory activities of synthesized compounds (**CM1-CM10**) were determined by a Homogeneous Time Resolved Fluorescence (HTRF) assay. The phosphorylation of a TK substrate triggered by MET kinase is measured in this method. Table 3 summarizes the inhibition results for **CM1-CM10** at three concentrations of 10, 25, and 50 μM. **CM9** significantly inhibited MET kinase with percent activities of 53.0 and 66.3% at 25 and 50 μM, respectively, while **CM7**, **CM8**, and **CM10** showed weaker inhibitory effects. In contrast, **CM1-CM6** were devoid of any effect against MET. Cabozantinib was also evaluated as a positive control with an IC_50_ value of 24.4 nM. Furthermore, the concentration-effect curve of **CM9** was also determined by HTRF assay and an IC_50_ value of 22.8 ± 3.9 µM was calculated (Figure 3).

**TABLE 3:** MET kinase inhibitory activity of synthetic compounds **CM1-CM10** determined by HTRF assay.

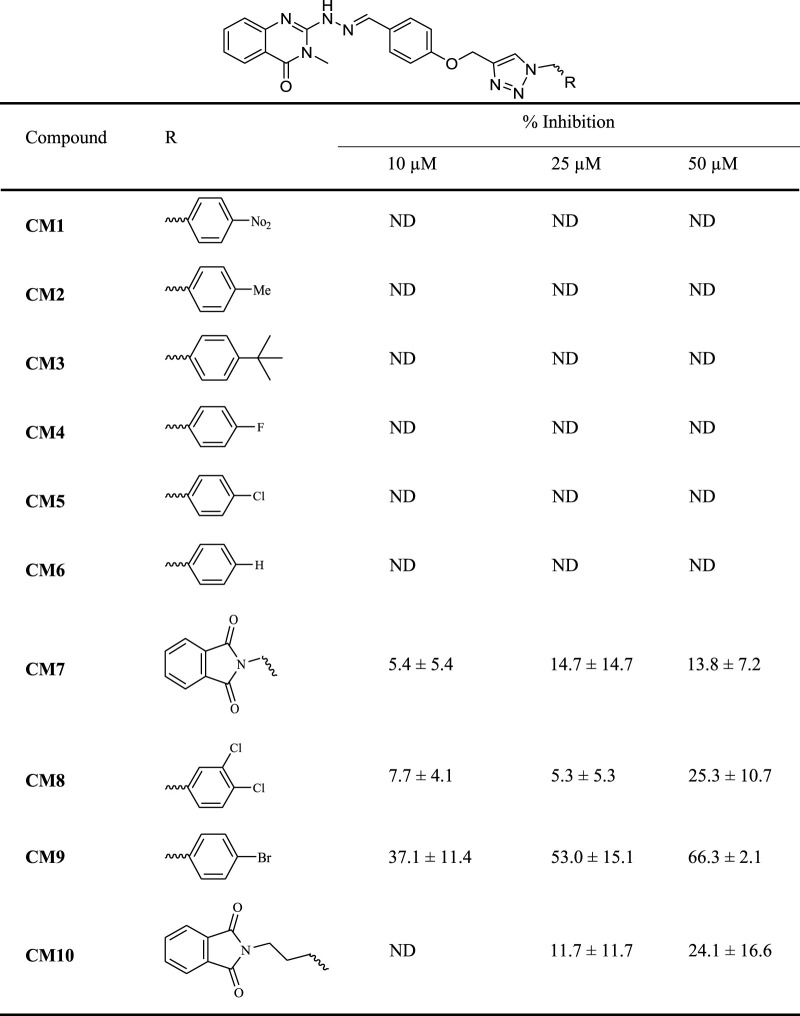


Values are the mean ± S.E.M. of 3–6 separate experiments. Crizotinib and cabozantinib were used as a positive controls with IC_50_ values 15.3 nM and 24.4 nM, respectively. ND: not determined.

### 3.4 Antiproliferative effect against cancer cells grown in three-dimensional cultures

**FIGURE 4:**
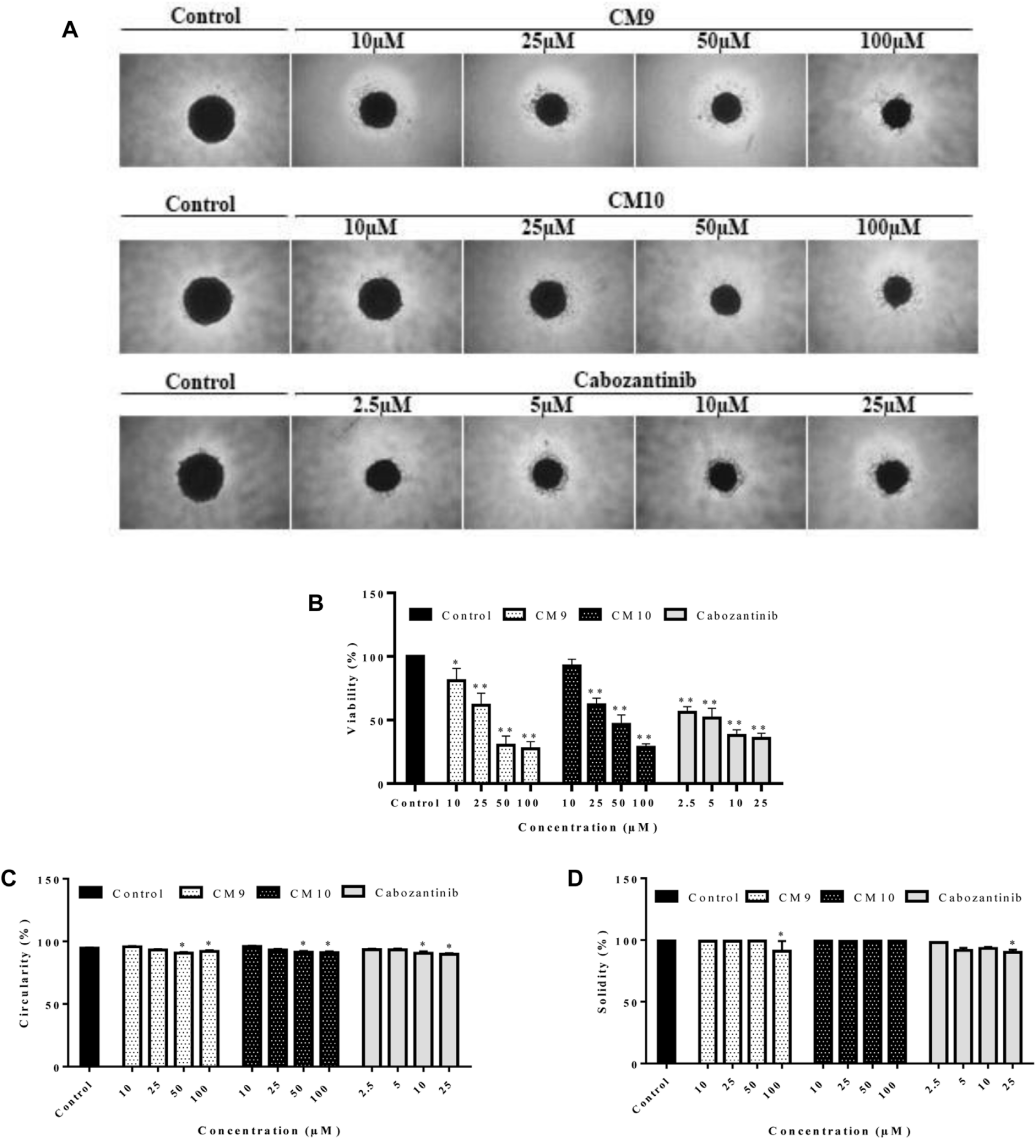
Growth inhibitory effects of synthesized compounds against cancer cells grown in three-dimensional spheroid cultures. Spheroids of EBC-1 cells were formed by agarose-based liquid overlay technique in 96-well plates. **(A)** Representative images of spheroids treated with **CM9** and **CM10** at 10, 25, 50, and 100 µM are shown. The images were captured with Nikon NIS-Elements imaging software. **(B)** Growth inhibitory effects of compounds against EBC-1 spheroids was measured by acid phosphatase (APH) assay. **(C)** Circularity and **(D)** solidity of 3D spheroids after drug treatment were measured by ImageJ software. Cabozantinib was also tested as a positive control. Data are presented as mean ± S.E.M. of 3-6 independent experiments. The difference with control was statistically significant at * (*p* < 0.05), ** (*p* < 0.01).

The growth inhibitory effects of active derivatives **CM9** and **CM10** were assessed in three-dimensional (3D) spheroid models. After formation of EBC-1 single spheroids with liquid overlay technique, treatment with different concentrations of **CM9** and **CM10** was performed. The growth inhibitory effects of selected derivatives was measured by acid phosphatase (APH) assay. There was a significant reduction in cell viability of spheroids treated with **CM9** and **CM10** derivatives compared to control in a dose-dependent manner (Figures 4A,B). Moreover, two spheroid indices including circulatory and solidity were also analyzed. The spheroid circulatory was dose dependently decreased, although this effect was less prominent in solidity analysis (Figures 4C,D).

### 3.5 Apoptosis induction measured by Hoechst 33258 staining

**FIGURE 5:**
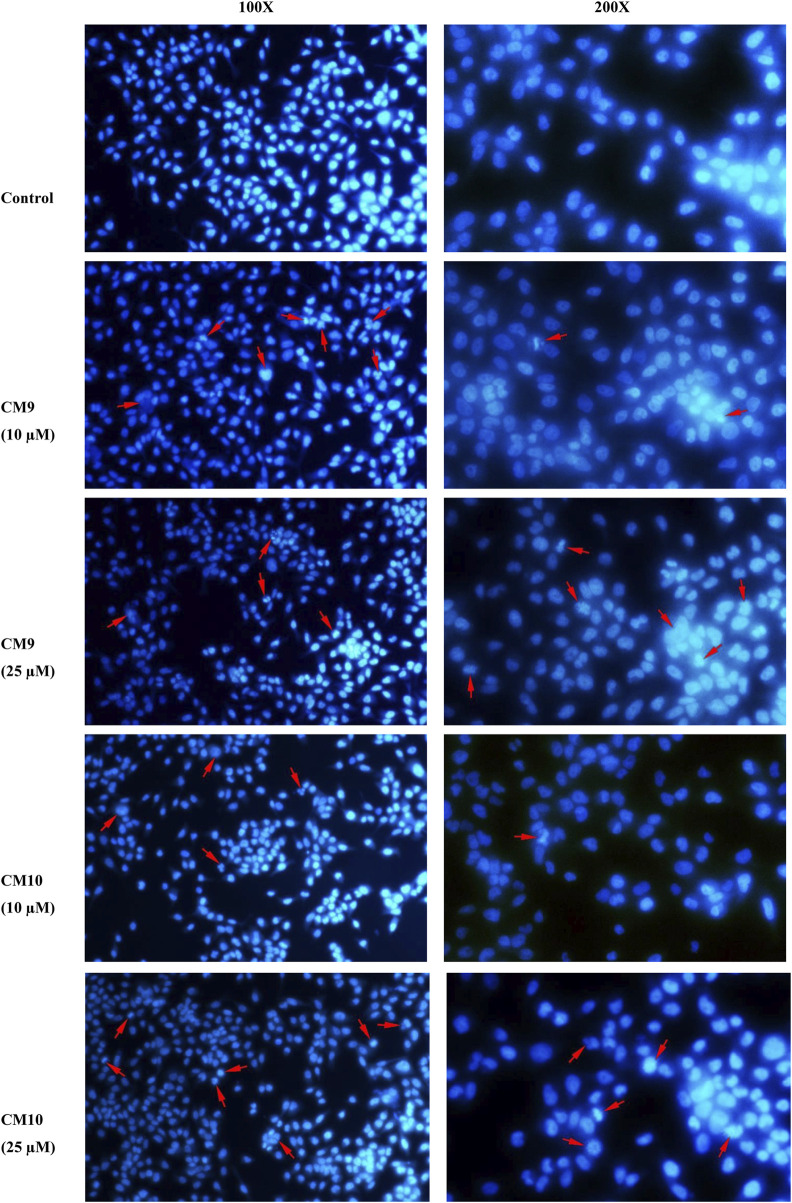
Apoptosis induction measurement by Hoechst 33258 staining assay. EBC-1 cells were seeded in 6-well plates and treated with different concentrations of test compounds for 72 h. After fixation with 4% cold paraformaldehyde (PFA) the cells were stained with 2.5 μg/ml Hoechst 33258 and imaged with a fluorescence microscope (Mag: 100X and 200X). A representative image is shown for each compound. Red arrows show apoptotic cells.

Considering the results obtained by the MTT assay, compounds **CM9** and **CM10** were selected for further investigation by Hoechst 33258 staining. Hoechst 33258 is a fluorescent DNA stain for apoptosis studies; Live cell nuclei are detected with a uniformly light blue emission, while apoptotic cell nuclei exhibit signs of apoptosis. As shown in Figure 5, EBC-1 cells exposed to 10 and 25 µM of test compounds exhibited the characteristic morphological changes of apoptotic nuclei, such as chromatin condensation, nuclear fragmentation and shrinkage in contrast to control cells with no apparent morphological changes. The results indicated that these agents induced apoptosis and might be considered as promising anticancer agents.

### 3.6 Kinase selectivity profile

In order to investigate the activity of compound **CM9**, as the most promising agent in this series, against other oncogenic kinases, we further assessed this derivative against a panel of 18 other protein kinases using a radiometric assay at the concentrations of 10 and/or 25 μM (Table 4).

**TABLE 4:** Measurement of the inhibitory effects of synthetic compound CM9 against a panel of oncogenic protein kinases.

Kinase name	Kinase inhibition (%)^a^	
	10 µM	25 µM
1) ABL1(h)	14	
2) ALK(h)	25	**51**
3) AXL(h)	28	**65**
4) KIT (c-Kit) (h)	0	
5) EGFR(h)	−2	
6) FGFR1(h)	39	**66**
7) FLT1 (VEGFR1) (h)	22	**82**
8) FLT3 (h)	24	47
9) FLT4 (VEGFR3)	**86**	
10) KDR (VEGFR2) (h)	0	
11) MAPK3 (ERK1) (h)	−4	
12) MAPK1(m) (ERK2)	3	
13) MET(h)	27	**58**
14) PDGFRA (PDGFRα) (h)	5	
15) PDGFRB (PDGFRβ) (h)	23	10
16) RET(h)	−12	
17) MST1R (RON) (h)	5	
18) ROS1	−25	
19) NTRK1 (TRKA) (h)	4	

^a^ Percent inhibition was measured at the concentrations of 10 and 25 μM by a radiometric assay. The values in bold indicate kinase inhibition percents of higher than 50%.

**FIGURE 6:**
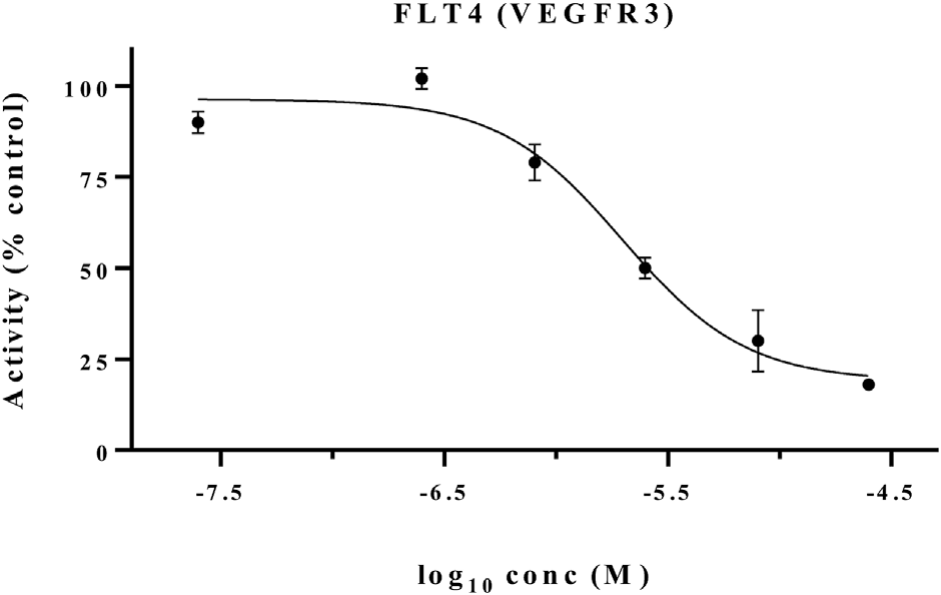
FLT4 (VEGFR3) kinase inhibitory activity of **CM9**. Kinase inhibition was examined by a Radiometric Assays. **CM9** inhibited FLT4 (VEGFR3) kinase activity with an IC_50_ of 5.01 µM.

Consistent with the HTRF results, **CM9** showed inhibitory activity against MET kinase also in this assay. Interestingly, this compound also demonstrated a considerable inhibitory potential against FLT4 (VEGFR3) by 86% at 10 μM. The concentration-effect curve was determined against this kinase, and the results are shown in Figure 6. As demonstrated in Table 4, **CM9** also exhibited inhibitory effect against ALK (Anaplastic lymphoma kinase), AXL, FGFR1 (Fibroblast growth factor receptors 1), and FLT1 (VEGFR1) with the inhibitory activities of 51%, 65%, 66%, and 82% at 25 μM, respectively. These results suggest that compound **CM9** is a promising multitarget RTK inhibitor.

### 3.7 *In silico* studies

#### 3.7.1 Molecular docking studies

**FIGURE 7:**
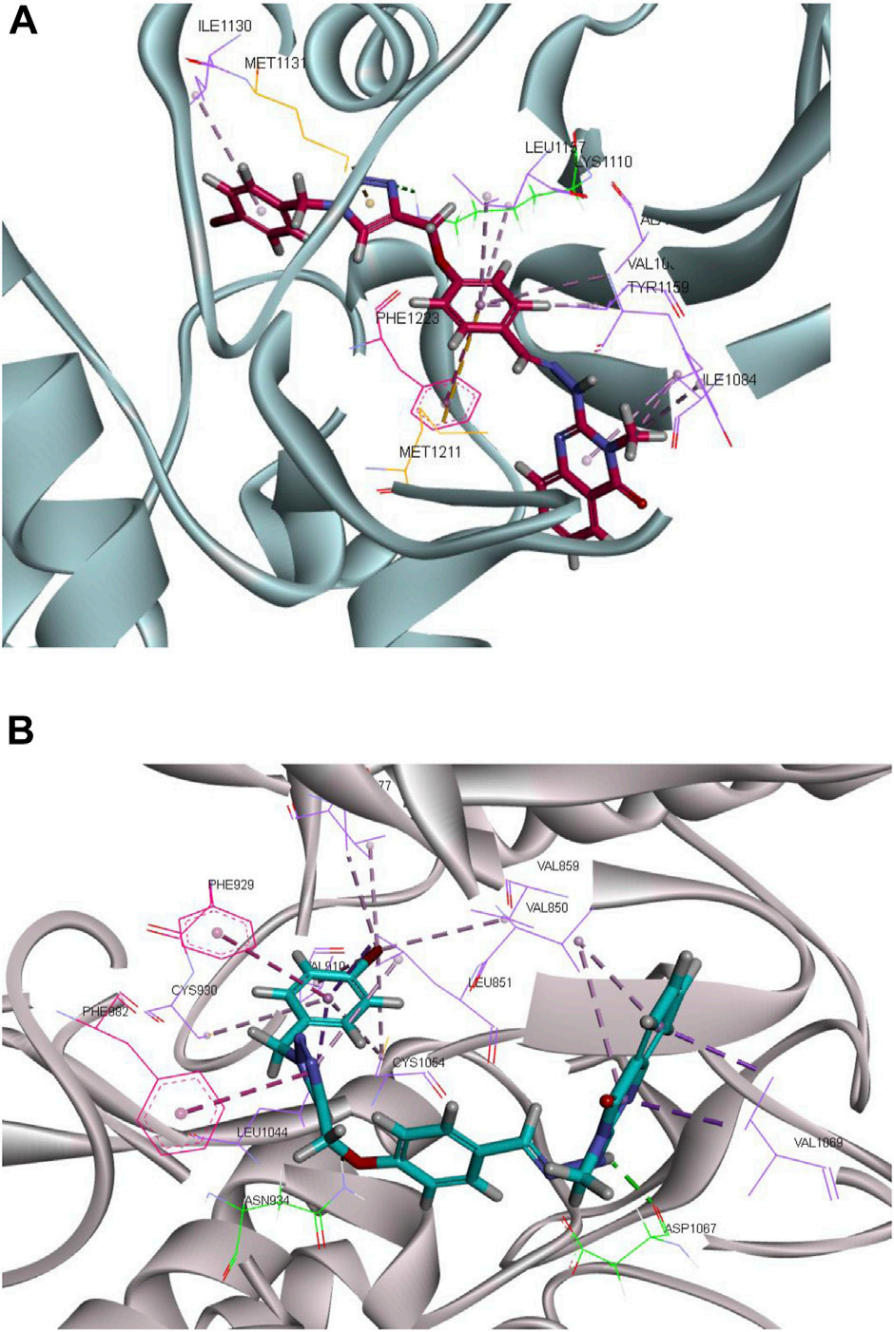
Molecular docking analysis of the interaction of **CM9** with MET receptor and established FLT4 (VEGFR3) homology model. Interactions and binding modes of **CM9** with MET **(A)** and VEGFR3 kinase **(B)** are shown. Van der Waals, hydrogen bond, pi–pi and pi–sulfur interactions were colored as light purple, light green, light pink and orange. Images were created by Discovery Studio Client v12.2.0.16349.

Molecular docking analysis was carried out in an attempt to evaluate the ability of synthesized compounds to interact with MET and FLT4 (VEGFR3) kinase using smina docking. The co-crystallized structure of MET (PDB code: 3LQ8) in complex with foretinib and the established FLT4 (VEGFR3) homology model were utilized for the docking study. The smina docking algorithm was evaluated using the re-docking of foretinib into the active site of MET kinase. The root-mean-square deviation (RMSD) value was 1.5 Å which is lower than the tolerable marginal value of 2 Å. Figure 7 illustrates docking interactions of the most potent compound (**CM9**) in the active site of MET and VEGFR3 kinase.

As shown in Figure 7A, compound **CM9** made one hydrogen bond interaction with Lys1110 in the kinase domain of MET through triazole ring. The phenyl ring of the methoxyphenyl linker participated in the pi-pi stacked interactions with Phe1223. Moreover, quinazoline and phenyl ring formed pi-alkyl interaction with Ile1130, Ala1108, Val1092, Leu1157, Ile1084, and Try1159. Phenyl and triazole rings made five pi-sulfur interactions with Met1131, Met1211, Lys1110, Glu1127, and Asp1222.

On the other hand, examining **CM9** binding mode with (FLT4) VEGFR3 demonstrated two hydrogen bond interactions with Asp1067 and Asn934, through NH of hydrazine and oxygen of methoxy linkers, respectively. Triazole and phenyl rings participated in the pi-pi interaction with Phe982, and Phe929, respectively. Moreover, quinazoline, triazole, and phenyl rings made pi-alkyl interactions with Val1069, Val850, Leu851, Ala877, Val910, Cys930, Leu1044, Cys1054, Val859, and Val927 (Figure 7B).

#### 3.7.2 Molecular dynamics simulation of CM9 with MET and VEGFR3 receptors

**FIGURE 8:**
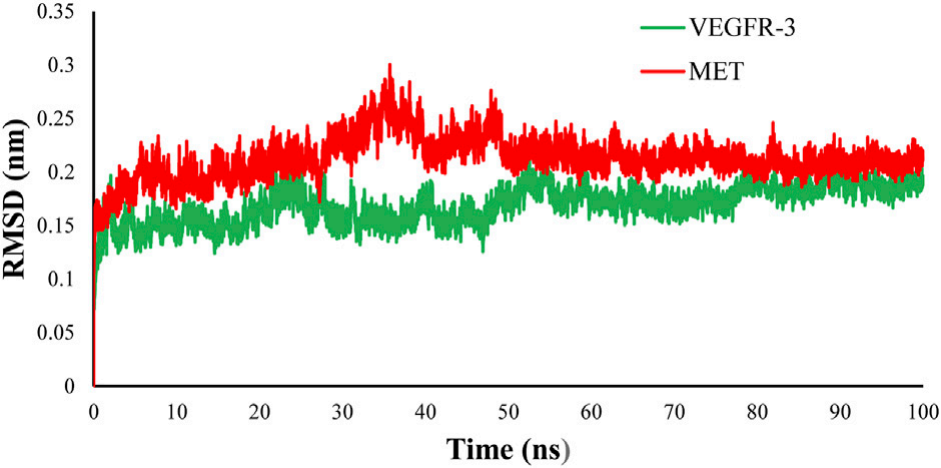
RMSD evaluation of complex of **CM9** with MET kinase (pdb:3LQ8) (red) and established FLT4 (VEGFR3) homology model (green). To resolve the MD equilibrium time range, the RMSD plot of the protein backbone atoms *versus* time for the interaction of the **CM9** with MET kinase (pdb:3LQ8) (red) and established VEGFR3 homology model (green) were investigated. The equilibrium time ranges of 45 and 60 ns were observed for complexes of **CM9** with MET and VEGFR3 kinases, respectively. The RMSD figure was drawn by Microsoft Excel 2010.

MD simulation was carried out for MET and FLT4 (VEGFR3) kinases in complex with the most potent compound **CM9**. To evaluate the conformational stability of the enzyme, the RMSD values were measured based on the alignment of backbone atoms of each frame *versus* the first frame against time over the whole course of simulations. The regular RMSD profile showed that **CM9** reached the equilibrium phase at 45 ns for MET and 60 ns for FLT4 (VEGFR3) complexes (Figure 8).

**FIGURE 9:**
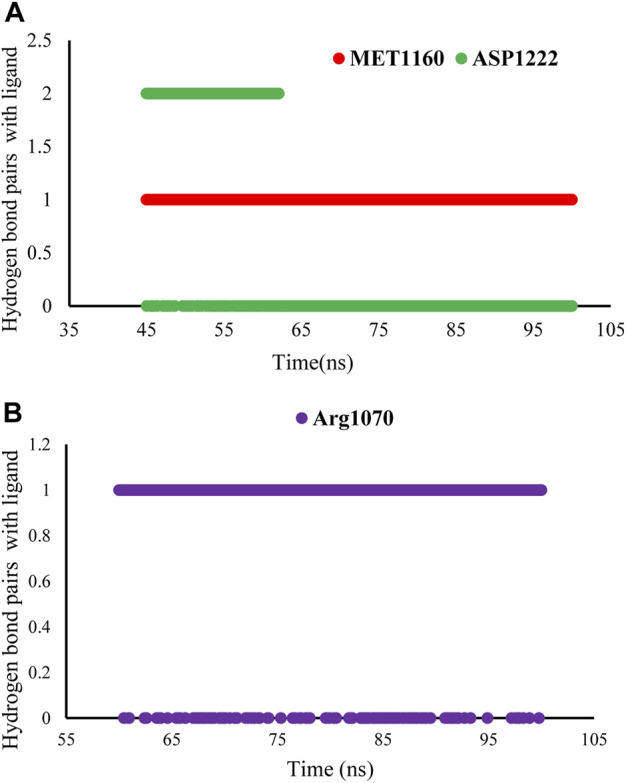
Stability of hydrogen bond interactions of compound **CM9** with MET and FLT4 (VEGFR3). The stability of hydrogen bond interactions of **CM9** derivative in the equilibrium time range and promising residues inside the active site of MET **(A)** and FLT4 **(B)** are shown.

In this study, we calculated the number of hydrogen bonds formed as a function of time between the **CM9** and amino acid residues in the active site of MET and FLT4 (VEGFR3) during the equilibrium time range of MD simulations. According to the obtained results, **CM9** made at least one hydrogen bond 95.82% of the time with MET kinase and one and two hydrogen bonds in 91.84% and 54.41% of the times with FLT4 (VEGFR3) kinase, respectively. Furthermore, a maximum of three and six hydrogen bonds were formed for MET and FLT4 (VEGFR3) kinase, respectively. Moreover, the stability of hydrogen bond interactions between the ligand CM9 and promising residues inside the active sites of MET and FLT4 in the equilibrium time range was shown in Figure 9.

**FIGURE 10:**
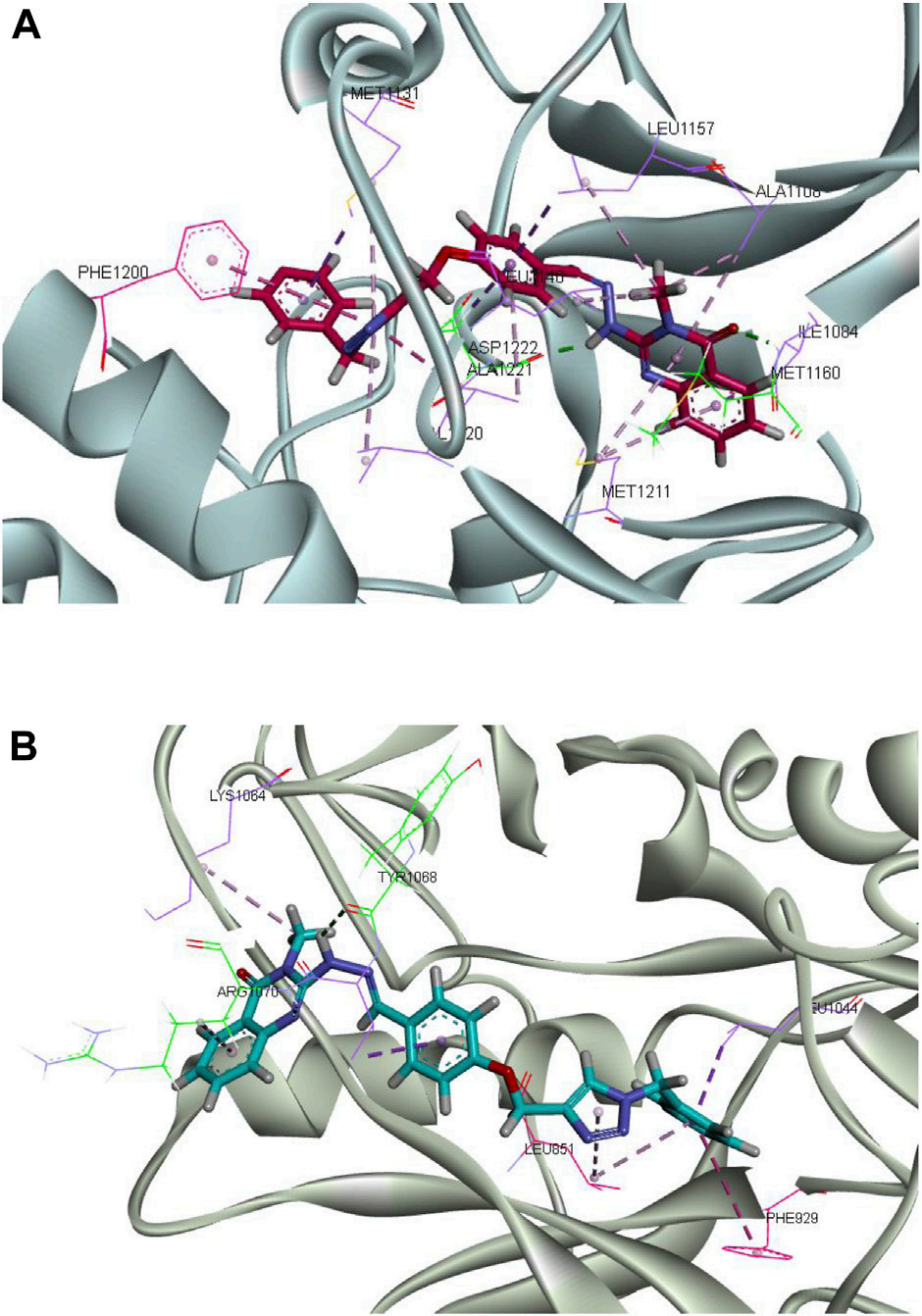
Molecular dynamics simulation analysis of the interactions of **CM9** with MET and FLT4 (VEGFR3). Molecular dynamics (MD) simulation analysis of **CM9** with MET **(A)** and FLT4 (VEGFR3) kinases **(B)** were perfrormed with Gromacs software. Van der Waals, hydrogen bond, pi–pi and pi–sulfur interactions were colored as light purple, light green, light pink and orange. Images were created by Discovery Studio Client v12.2.0.16349.

The clustering analyses of complex **CM9** with both targets were carried out, and the result showed that the percent of the population in cluster 1 was 97.64% with MET and 97.25% with FLT4 (VEGFR3) kinase. The representative frame of cluster 1 of each complex is shown in Figure 10. The interaction patterns of representative frames of all the clusters have been shown in the Supplementary Material.

Analysis of interaction of **CM9** with MET kinase showed two critical hydrogen bonds from carbonyl of quinazoline ring with Met1160 and a hydrogen bond from NH of the hydrazide linker with Asp1222. Also, two important pi-pi interactions were made between the triazole ring with Phe1200 and Ala1221. Moreover, **CM9** made hydrophobic interactions with Ile1084, Ala1108, Met1211, Leu1157, Val1220, Met1131 and Ala1221, see Figure 10A.

Moreover, as shown in Figure 10B, the complex of **CM9** with FLT4 (VEGFR3) kinase showed two hydrogen bonds from N atom of quinazoline with Arg1070 and N atom of hydrazine linker with Tyr1068. Phenyl rings participated in pi-pi stacked interaction with Phe929, and methoxyphenyl ring made amide pi-pi stacked interaction with Leu851. Furthermore, there was hydrophobic interaction from the common substructure of the compound with Arg1070, Lys1064, Val1069, Leu851, and Leu1044.

#### 3.7.3 MM-PBSA analysis

MM-PBSA approach was utilized for the calculation of the binding energies and ligand contributions of MET and FLT4 (VEGFR3) kinases in complex with the most potent compound **CM9.** The potent compound inside the mentioned proteins exhibited the potent mean values of average binding energies and ligand binding energy contributions -127.614 and -168.6113 for MET and -140.645 and -148.4372 kJ/mol for FLT4, respectively. However, the energy analysis of CM9 complex in both proteins has been shown in Table 5.

**TABLE 5:** The binding energy and energy contribution of ligand CM9 inside the active site of MET and FLT4 obtained from MMPBSA analysis in kJ/mol.

	Energy (MET) (kJ/mol)	Energy (FLT4) (kJ/mol)
Van der Waal energy	−285.260 ± 12.278	−252.696 ± 10.873
Electrostattic energy	−52.178 ± 9.918	−44.413 ± 11.507
Polar solvation energy	243.885 ± 19.192	191.590 ± 18.692
SASA energy	−34.061 ± 2.111	−35.126 ± 2.523
Binding energy	−127.614 ± 17.434	−140.645 ± 16.169
CM9	−168.6113 ± 0.7882	−148.4372 ± 0.7555

#### 3.7.4 Physicochemical properties

The SwissADME web tool was applied to predict pharmacokinetics properties and drug-likeness of all compounds (Daina et al., 2017). Most compounds presented acceptable molecular properties (Table 6).

**TABLE 6:** Physicochemical properties and drug-likeness prediction of designed compounds.

Compounds Physicochemical properties	CM1	CM2	CM3	CM4	CM5	CM6	CM7	CM8	CM9	CM10
MW (g/mol)	510.50	479.53	521.61	483.5	499.95	465.51	534.53	534.40	544.40	548.55
Number of rotatable bonds	9	8	9	8	8	8	8	8	8	9
Number of H-bond acceptors	8	6	6	7	6	6	8	6	6	8
Number of H-bond donors	1	1	1	1	1	1	1	1	1	1
MLog Po/w	2.62	3.62	4.20	3.78	3.88	3.42	2.61	4.35	3.98	2.40
MR	143.87	140.02	154.32	135.01	140.06	135.05	150.36	145.07	142.75	155.17
TPSA (Å^2^)	145.04	99.22	99.22	99.22	99.22	99.22	136.60	99.22	99.22	136.60

## 4 Discussion

In this study, 10 novel quinazolinone hydrazine triazole derivatives were synthesized and evaluated for MET inhibitory effect in cell-free and cell-based assays. Among them, compound **CM9** bearing *p*-bromo benzyl pendant on the triazole ring exhibited the highest MET inhibitory activity and anti-proliferative effects towards MET-amplified lung cancer cells. This derivative together with **CM10** bearing phetalimidemoiety also exhibited growth inhibitory effects against three-dimensional spheroid cultures of cancer cells and induced characteristic morphological changes of apoptosis. Data from a kinase selectivity profile assessment showed that compound **CM9** is able to inhibit other important oncogenic kinases such as FLT4 (VEGFR3). Computational studies supported our experimental observations and showed critical interactions between synthesized derivatives and target kinases.

In an attempt to evaluate the anticancer potential of synthesized compounds, antiproliferative effects were assessed against four cancer cell lines, including lung **(**EBC-1 and A549), colorectal (HT-29) and glioblastoma (U-87MG) cells measured using MTT assay in monolayer cell cultures. The lowest IC_50_ value was observed for compound **CM9** (8.6 µM) against the MET amplified EBC-1 cell line. It is interesting to note that EBC-1 are dependent on MET oncogene for proliferation and survival and are considered a good model for testing the MET inhibitory potential of different compounds (proteinatlas.org, 2022; cansarblack.icr.ac.uk, 2022). Moreover, another derivative, **CM10**, displayed moderate growth inhibitory effects in the EBC-1 and HT-29 cells with IC_50_ values of 18.0 ± 5.6 and 18.4 ± 2.3 µM, respectively.

According to the results of MET kinase inhibition and MTT assay, **CM9** bearing para-bromophenyl moiety seems to be the most promising agent. As for the structure-activity relationships (SAR), there seems to be a strong relationship between the nature and position of the phenyl substitution linked to triazole ring and MET kinase inhibition; the introduction of bulky electron withdrawing groups (EWG) at para position of benzyl linked to triazole ring enhances the activity of compounds as represented in compound **CM9** bearing *p*-bromo benzyl pendant. In addition, compounds with two-EWG at meta- and para-positions of benzyl moiety were better than mono-EWGs substituted derivatives; compound **CM8** with two chlorine groups at 3, 4-position of benzyl pendant demonstrated intermediate inhibitory activity, while its para-chlorinated counterpart **CM5** was almost inactive. Finally, the presence of bulky lipophilic groups like phetalimide on the triazole ring showed modest influence on MET inhibitory potential of compounds. For instance, **CM10** and **CM7** showed intermediate inhibitory activities.

It has been recently recognized that three-dimensional cellular models are powerful tools offering reliable platforms for *in vitro* drug screenings. Compared to conventional two-dimensional cultures, three-dimensional models can more closely mimic features of *in vivo* human solid tumors, such as their gene expression patterns, complexity, heterogeneity, as well as drug resistance (Firuzi et al., 2019). In this context, the growth inhibitory effect of the most promising antiproliferative agents, **CM9** and **CM10,** was evaluated in EBC-1 cells grown in 3D cultures. It was observed that the spheroid viability as well as their physical properties, including circularity and solidity, were decreased after both **CM9** and **CM10** exposure in a dose-dependent manner.

Moreover, the morphological studies performed using DNA staining with Hoechst 33258 confirmed the apoptotic induction effect of **CM9** and **CM10** on EBC-1 cells. These compounds displayed typical apoptotic features such as nuclear shrinkage and fragmentation.

The inhibitory potentials of the most promising agent, **CM9**, was assessed against a panel of 18 well-known oncogenic kinases. This derivative showed considerable inhibitory activity against FLT4 (VEGFR3) receptor tyrosine kinase, in addition to MET kinase. Targeting the members of VEGFR families, noticeably VEGFR1, VEGFR2 and VEGFR3, have been evaluated as potential antiangiogenic therapies. In particular, VEGFR-3 plays a vital role in the progression of lymphangiogenesis, in addition to angiogenesis, promoting tumor cell invasion and metastasis. Therefore, the development of drugs targeting the FLT4 (VEGFR3) signaling pathway may be therapeutically beneficial in cancer management (Hsu et al., 2019). **CM9** also exhibited considerable potency against ALK (Anaplastic lymphoma kinase), AXL, FGFR1 (Fibroblast growth factor receptors 1), and FLT1 (VEGFR1). Based on our findings, **CM9** represents a promising kinase inhibitor. Clearly, due to a high degree of sequence and structural similarity in the kinase domain of RTKs, a large numbers of kinase inhibitors have an expected cross-reactivity within the kinase family (Pottier et al., 2020). Until now, 55 small molecule compounds targeting kinase proteins, especially RTKs, have received FDA approval for indications in oncology, and among them, at least 25 agents are multi-targeted, inhibiting several protein kinases (Roskoski, 2020). Cabozantinib, as an approved RTK inhibitor, for example, is effective in targeting a broad range of RTKs, including MET, VEGFR-1/2/3, RET (Rearranged during transfection), FLT3, AXL, and c-KIT (Viola et al., 2013).

Another important point to consider about multi-target agents is the drug resistance issue that appears to be a more severe problem for single-target drugs. In this regard, multi-target drugs have generally shown higher efficacy compared to single-target drugs in overcoming drug resistance (Raghavendra et al., 2018; Desai and Small, 2019). The interactions of compound **CM9** was also evaluated with MET and FLT4 (VEGFR3) kinases by docking analysis. **CM9** in the active site of MET kinase made an important hydrogen bond interaction with Lys1110 through triazole ring and a key pi-pi stacked interactions with Phe1223 from the phenyl ring of methoxyphenyl linker. Moreover, hydrophobic interactions through phenyl and triazole rings with residues in hydrophobic pocket may cause the higher inhibitory potential of **CM9**. In addition, docking studies of **CM9** with VEGFR3 demonstrated two hydrogen bond interactions with Asp1067 and Asn934 and two pi-pi interaction with Phe982, and Phe929.

In order to better understand the stability of the interactions of compound **CM9** with the active site residues, an MD simulation was also performed. It was observed that **CM9** makes two critical hydrogen bonds with Met1160 and Asp1221 in the kinase domain of MET. Also, two important pi-pi interactions were made between the triazole ring with Phe1200 and Ala1221, while hydrophobic interactions were made with hydrophobic residues. Moreover, **CM9** with FLT4 (VEGFR3) kinase showed two hydrogen bonds with Arg1070 and Tyr1068 and two pi-pi interactions with Phe929 and Leu851 and broad hydrophobic interactions with the active site of FLT4 (VEGFR3).

Hence, the findings of the computational analysis showed the formed important interactions from different part of **CM9** structure with key residues in the active site of targets may justify the inhibitory activities of this agent against MET and FLT4 (VEGFR3) kinases.

Finally, the evaluation of drug-like properties such as log P, molecular weight, number of hydrogen bond donors and acceptors, and TPSA were also performed on the basis of Lipinski’s rule of five. According to the predictions, all synthesized molecules were in accordance with Lipinski’s rule of five with no violations.

In this study we provide cell based and cell free assays as well as *in silico* studies to evaluate the MET inhibitory effects of 10 novel quinazolinone hydrazine triazole derivatives. The considerable antiproliferative effect of **CM9** and **CM10** derivatives against cancer cells was confirmed. The *In vitro* enzymatic assays results suggest **CM9** bearing *p*-bromo benzyl pendant on the triazole ring as a promising tyrosine kinase inhibitor especially against MET and FLT4 (VEGFR3). Eventually, important structural features for the interactions of **CM9** with MET and FLT4 (VEGFR3) kinases verified by Molecular docking and molecular dynamics simulation studies. These novel quinazolinone derivatives present promising anticancer agents with kinase targeting potential.

## Data Availability

The original contributions presented in the study are included in the article/Supplementary Materials, further inquiries can be directed to the corresponding authors.
