# Supplementary material for: Study of the anticancer effect of new quinazolinone hydrazine derivatives as receptor tyrosine kinase inhibitors
Source: Front Chem. 2022 Nov 17;10:969559. doi: 10.3389/fchem.2022.969559 (PMC9713320; doi:10.3389/fchem.2022.969559)
Supplement: Supplementary file 1 [file DataSheet1.PDF]

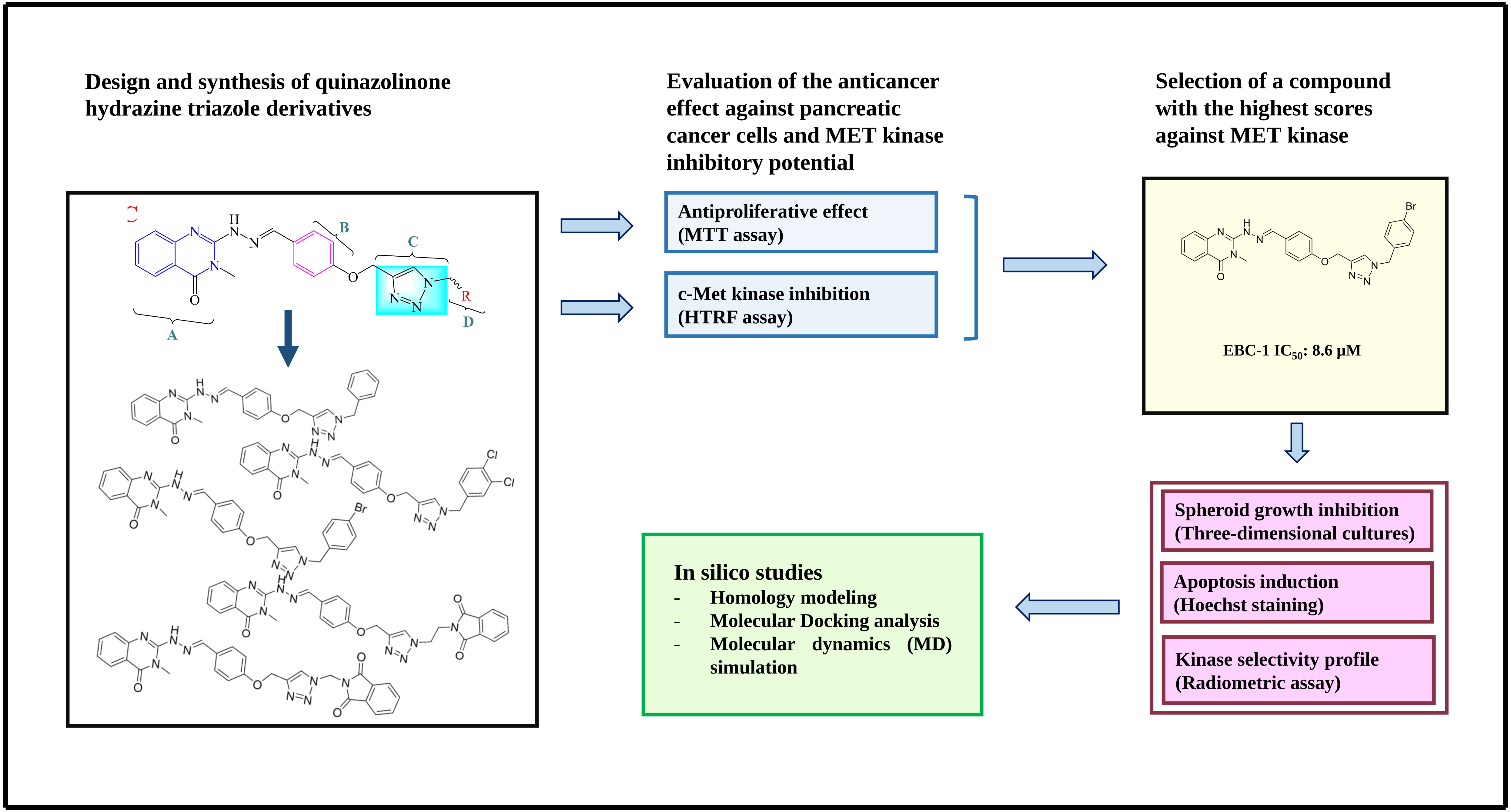
Design and synthesis of quinazolinone hydrazine triazole derivatives
Evaluation of the anticancer effect against pancreatic cancer cells and MET kinase inhibitory potential
Selection of a compound with the highest scores against MET kinase
A
B
C
D
R
Antiproliferative effect (MTT assay)
c-Met kinase inhibition (HTRF assay)
EBC-1 IC50: 8.6 µM
Spheroid growth inhibition (Three-dimensional cultures)
Apoptosis induction (Hoechst staining)
Kinase selectivity profile (Radiometric assay)
In silico studies
- Homology modeling
- Molecular Docking analysis
- Molecular dynamics (MD) simulation
