# Supplementary material for: Study of the anticancer effect of new quinazolinone hydrazine derivatives as receptor tyrosine kinase inhibitors
Source: Front Chem. 2022 Nov 17;10:969559. doi: 10.3389/fchem.2022.969559 (PMC9713320; doi:10.3389/fchem.2022.969559)
Supplement: Supplementary file 2 [file DataSheet2.docx]

**Supplementary Data**

**1. ^1^H-NMR and ^13^C-NMR Spectrum of synthetic compounds (CM1-CM10):**


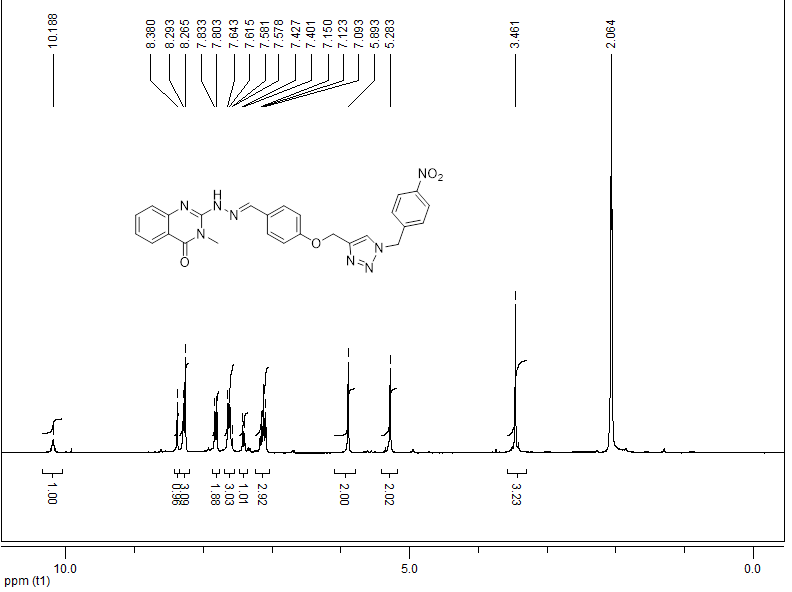


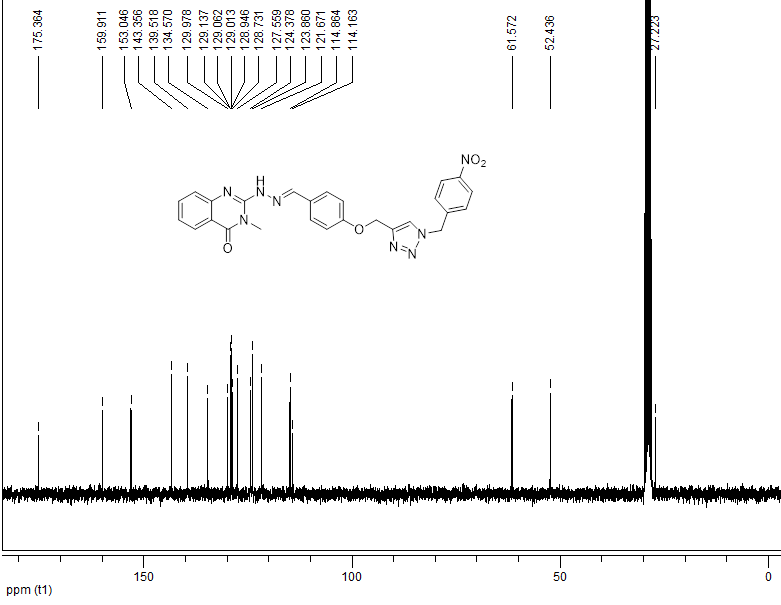


**9a**) **^1^H-NMR & ^13^C-NMR:** (*E*)-3-methyl-2-(2-(4-((1-(4-nitrobenzyl)-1H-1,2,3-triazol-4-yl)methoxy)benzylidene)hydrazineyl)quinazolin-4(3H)-one


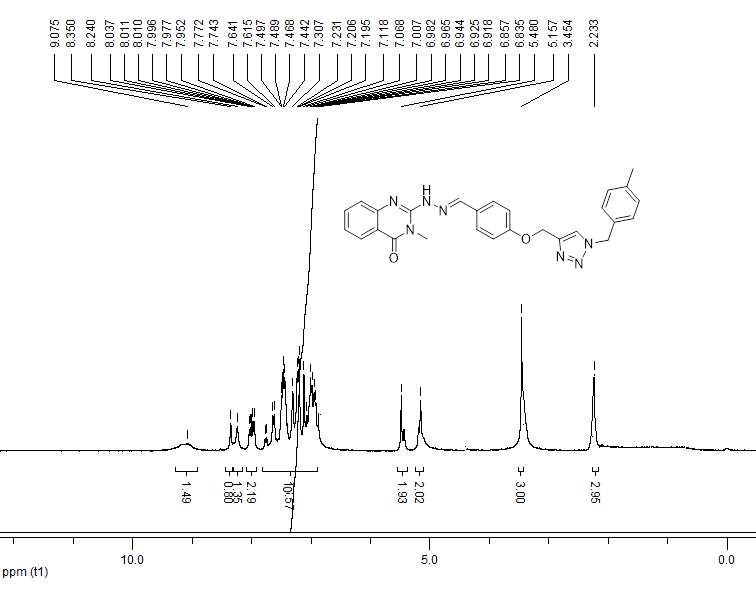


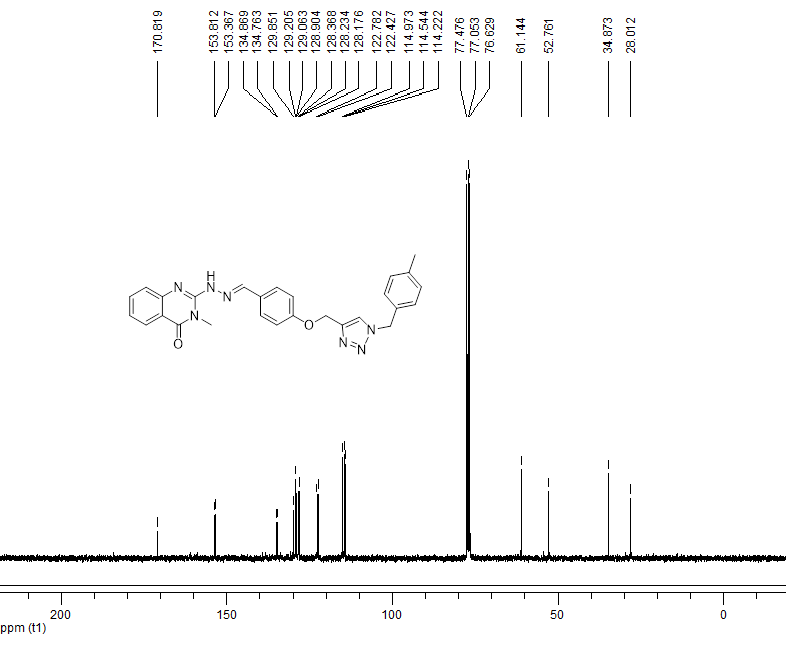


**9b**) **^1^H-NMR & ^13^C-NMR:** (*E*)-3-methyl-2-(2-(4-((1-(4-methylbenzyl)-1H-1,2,3-triazol-4-yl)methoxy)benzylidene)hydrazineyl)quinazolin-4(3H)-one


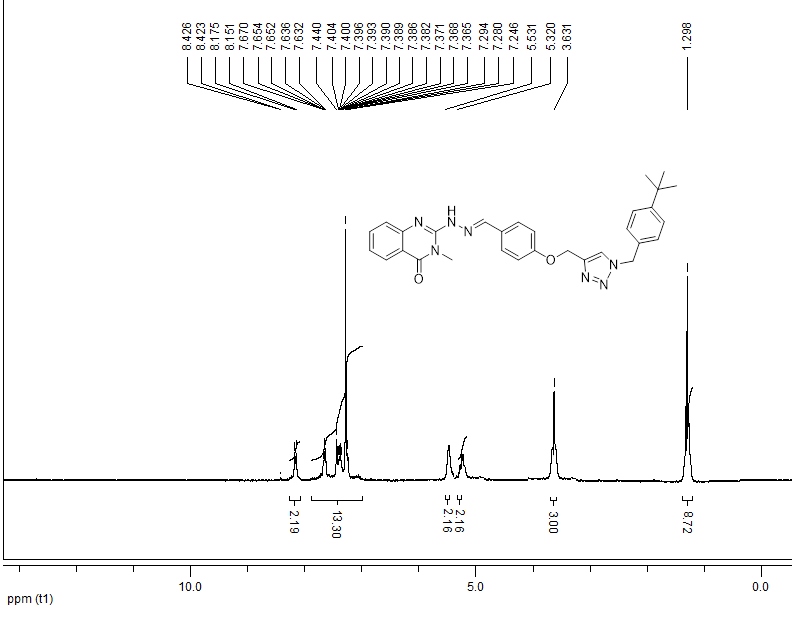


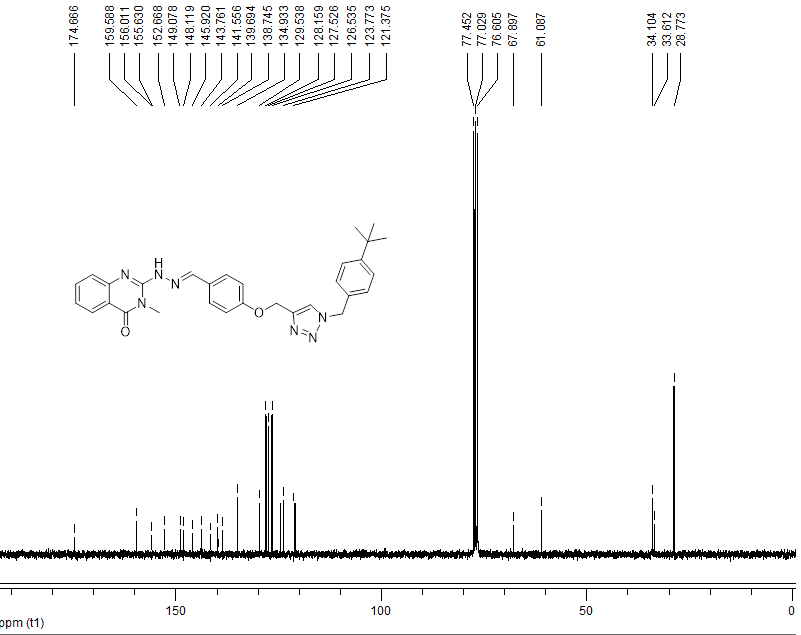


**9c**) **^1^H-NMR & ^13^C-NMR:** (*E*)-2-(2-(4-((1-(4-(tert-butyl)benzyl)-1H-1,2,3-triazol-4-yl)methoxy)benzylidene)hydrazineyl)-3-methylquinazolin-4(3H)-one


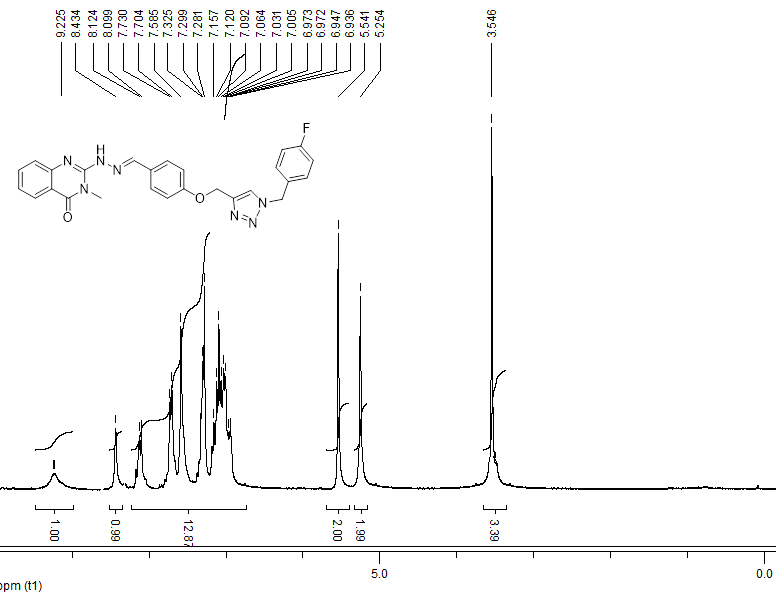


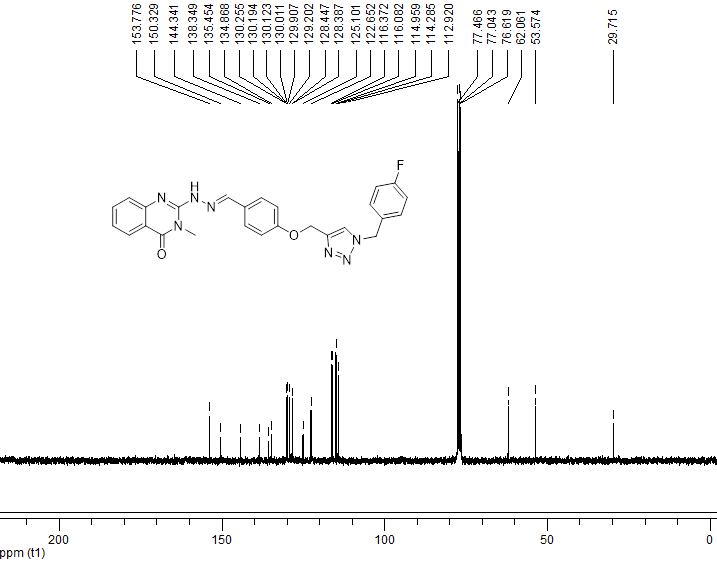


**9d**) **^1^H-NMR & ^13^C-NMR:** (*E*)-2-(2-(4-((1-(4-fluorobenzyl)-1H-1,2,3-triazol-4-yl)methoxy)benzylidene)hydrazineyl)-3-methylquinazolin-4(3H)-one


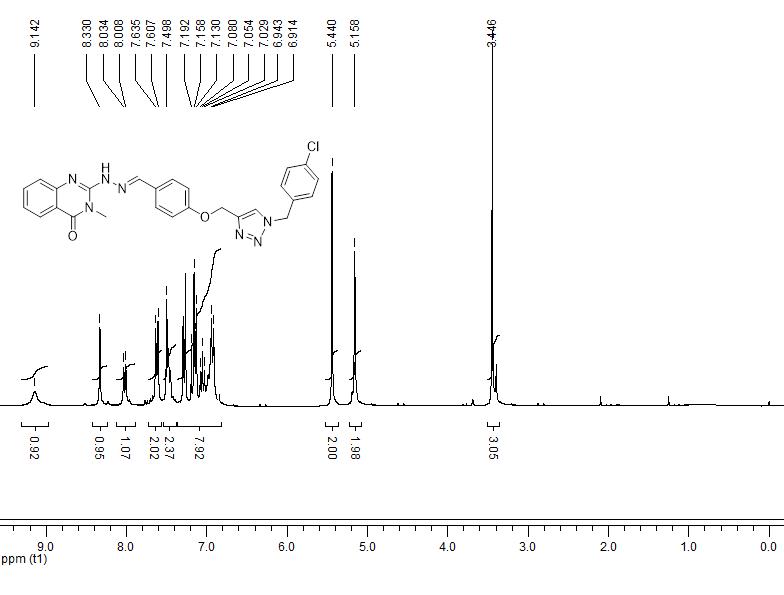


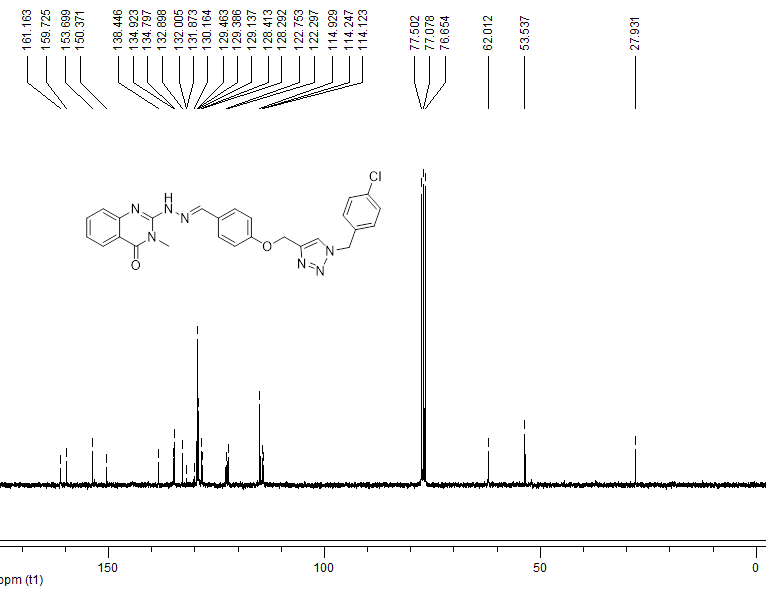


**9e**) **^1^H-NMR & ^13^C-NMR:** (E)-2-(2-(4-((1-(4-chlorobenzyl)-1H-1,2,3-triazol-4-yl)methoxy)benzylidene)hydrazineyl)-3-methylquinazolin-4(3H)-one


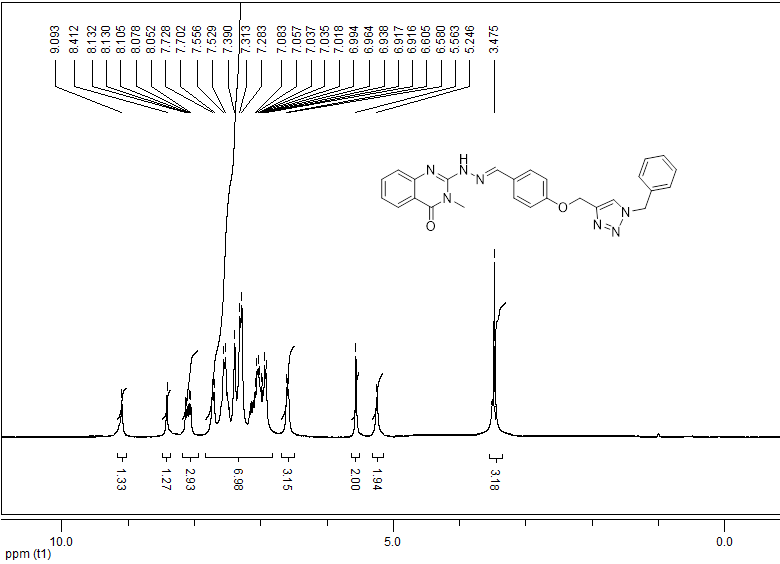


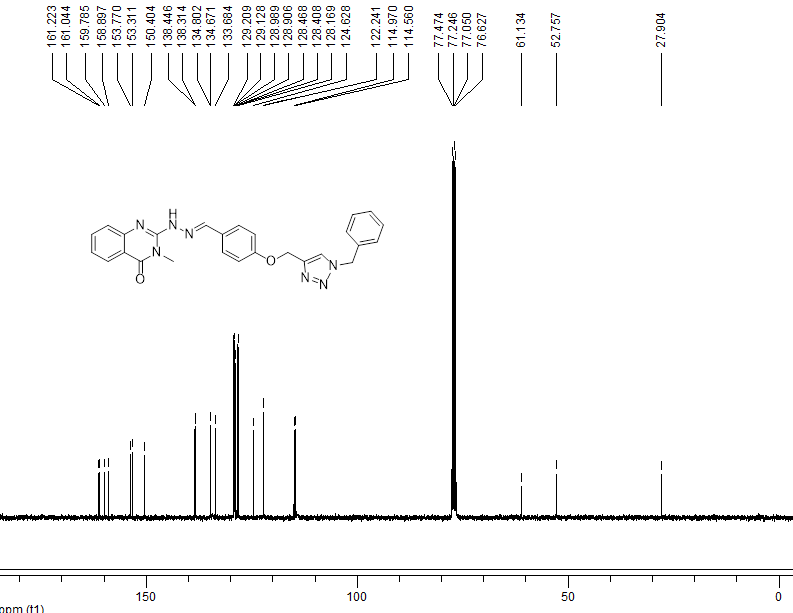


**9f**) **^1^H-NMR & ^13^C-NMR:** (E)-2-(2-(4-((1-benzyl-1H-1,2,3-triazol-4-yl)methoxy)benzylidene)hydrazineyl)-3-methylquinazolin-4(3H)-one


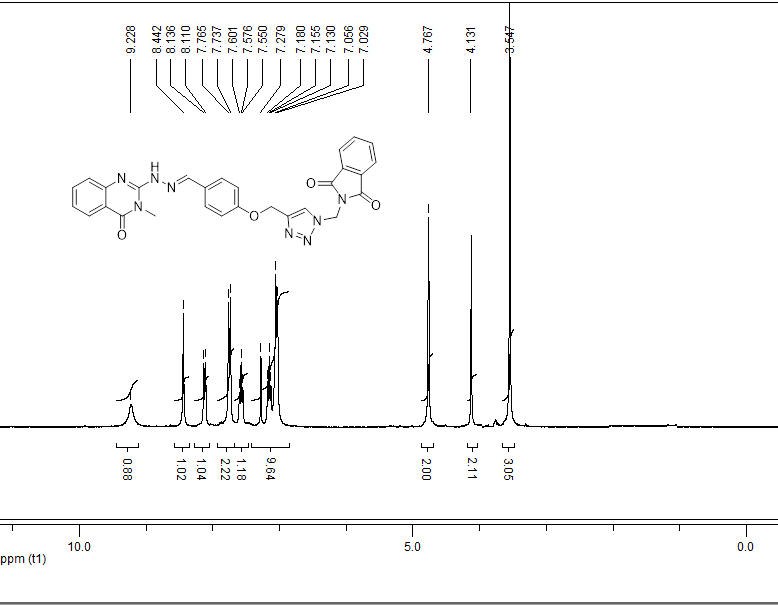


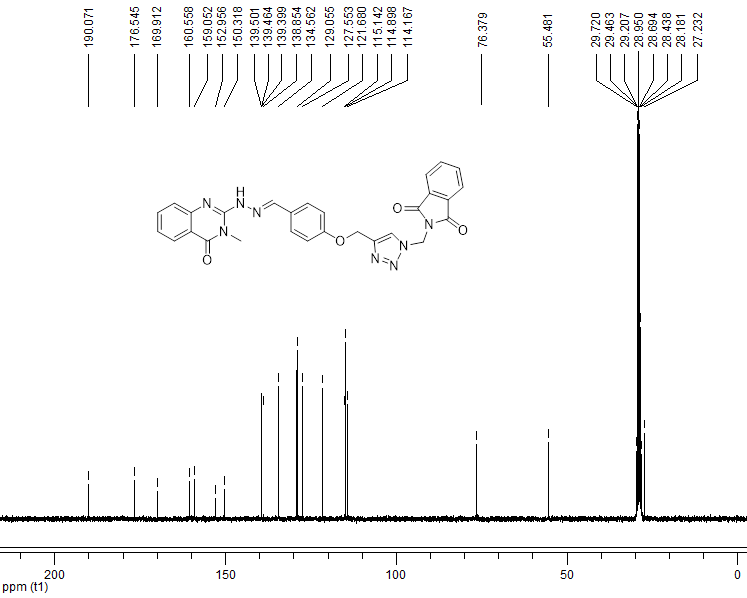


**9g**) **^1^H-NMR & ^13^C-NMR:** (*E*)-2-((4-((4-((2-(3-methyl-4-oxo-3,4-dihydroquinazolin-2-yl)hydrazineylidene)methyl)phenoxy)methyl)-1H-1,2,3-triazol-1-yl)methyl)isoindoline-1,3-dione


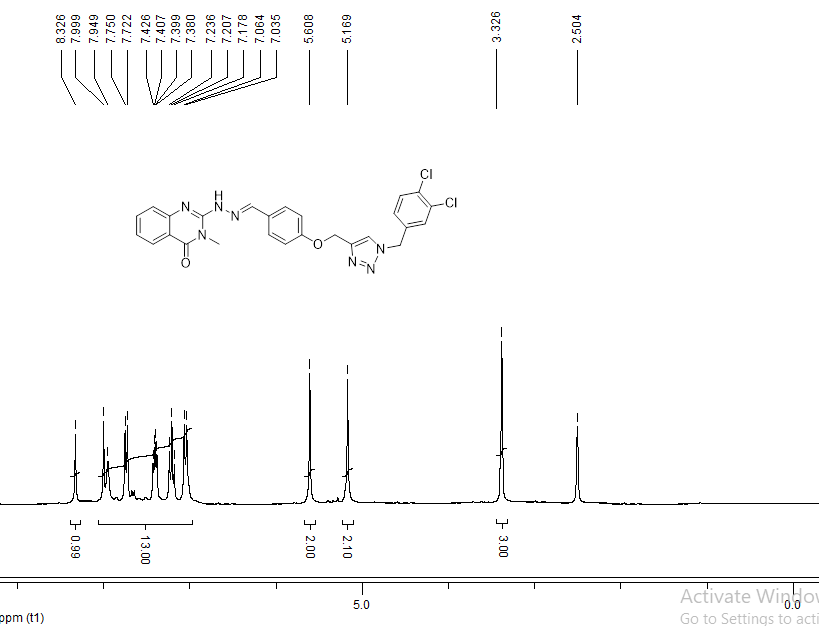

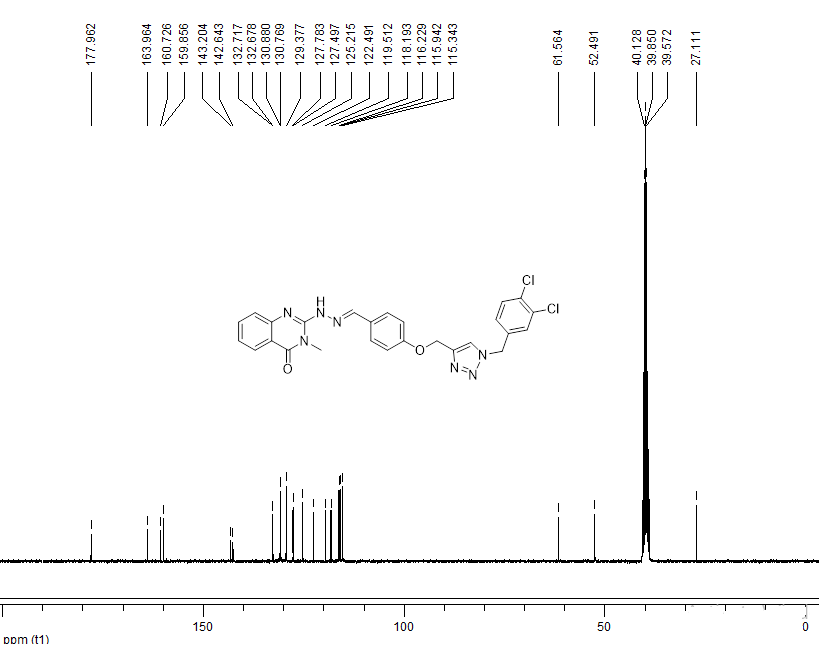


**9h**) **^1^H-NMR & ^13^C-NMR:** (*E*)-2-(2-(4-((1-(3,4-dichlorobenzyl)-1H-1,2,3-triazol-4-yl)methoxy)benzylidene)hydrazinyl)-3-methylquinazolin-4(3H)-one

**
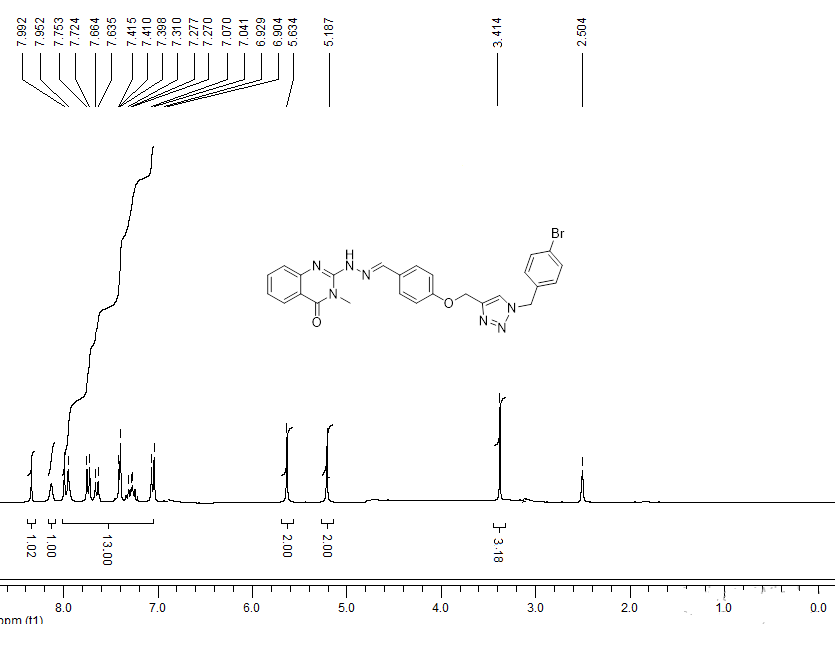

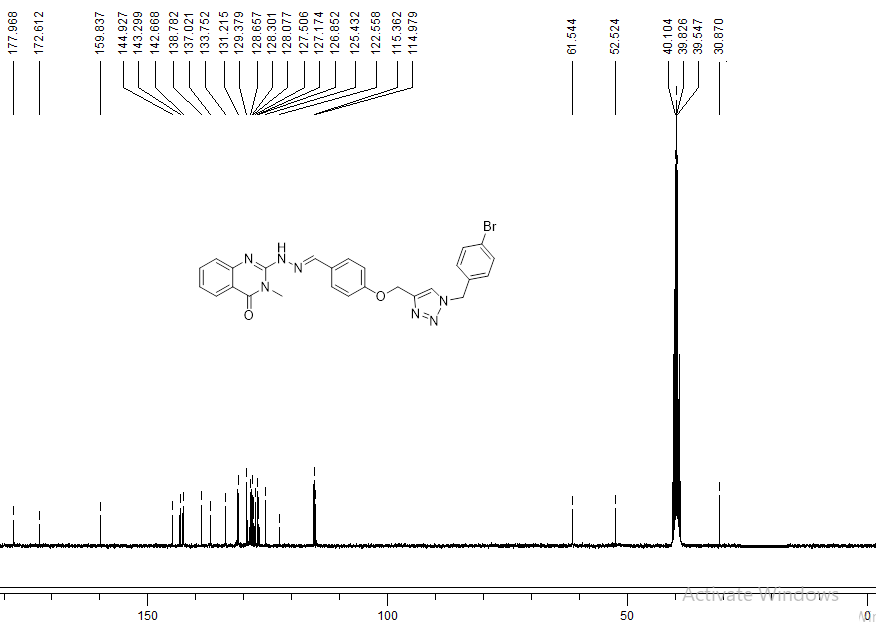
**

**9i**) **^1^H-NMR & ^13^C-NMR:** (*E*)-2-(2-(4-((1-(4-bromobenzyl)-1H-1,2,3-triazol-4-yl)methoxy)benzylidene)hydrazinyl)-3-methylquinazolin-4(3H)-one


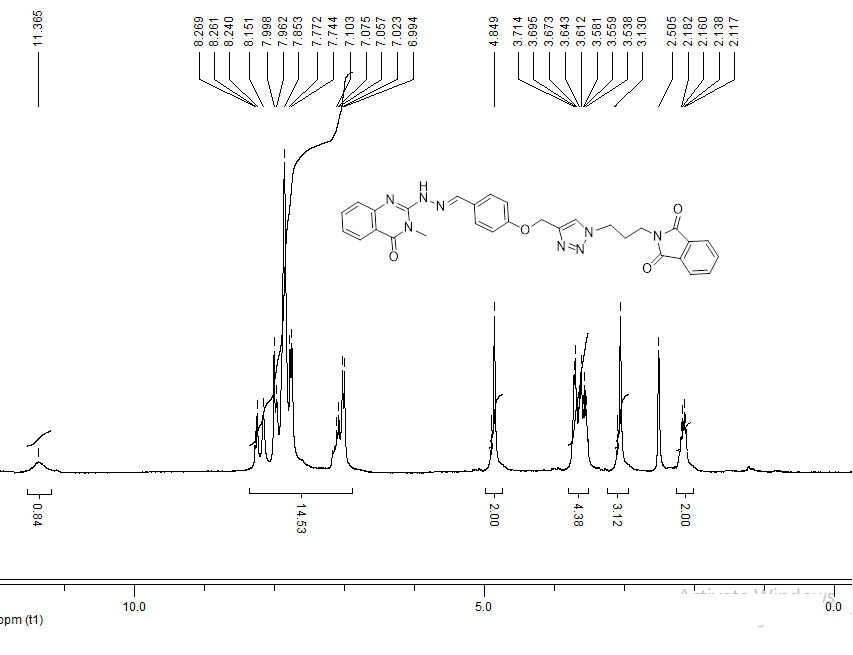
**
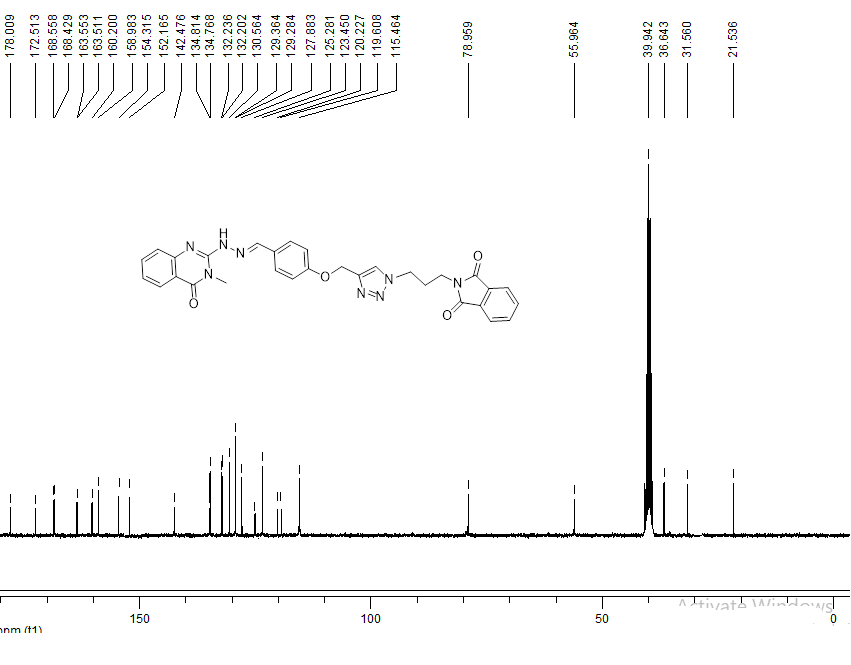
**

**9j**) **^1^H-NMR & ^13^C-NMR:** (*E*)-2-(2-(4-((4-((2-(3-methyl-4-oxo-3,4-dihydroquinazolin-2-yl)hydrazono)methyl)phenoxy)methyl)-1H-1,2,3-triazol-1-yl)ethyl)isoindoline-1,3-dione

**Cluster analysis:**

**Cluster analysis of CM9 with c-Met kinase**

| Cluster 1  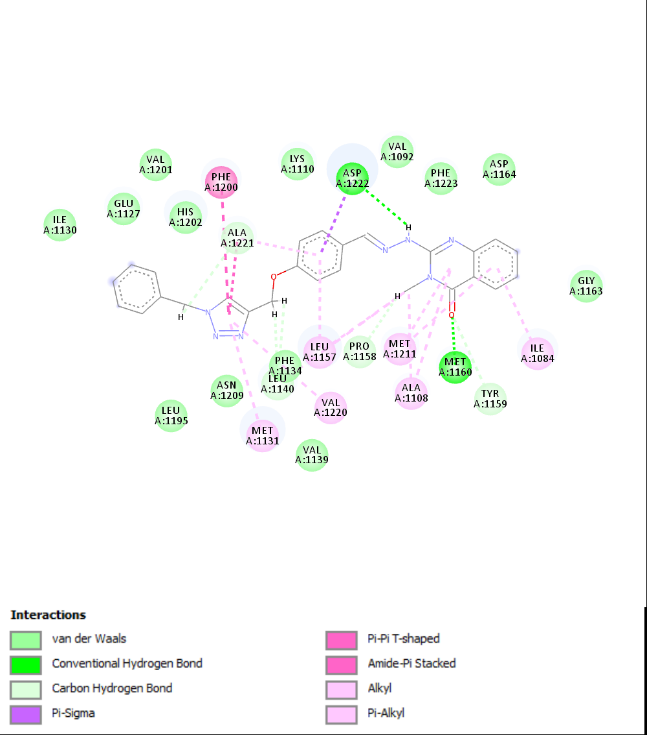 | Cluster2  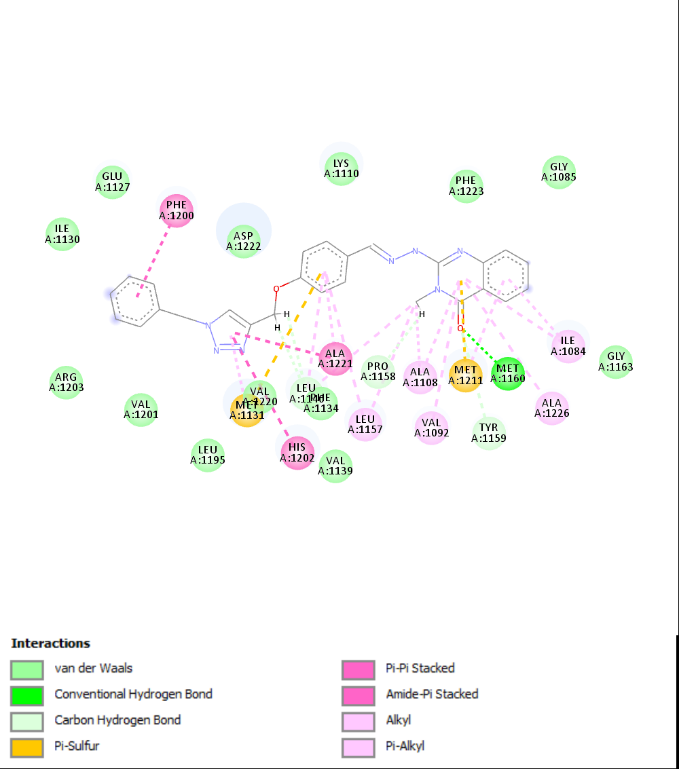 |
| --- | --- |
| Cluster 3  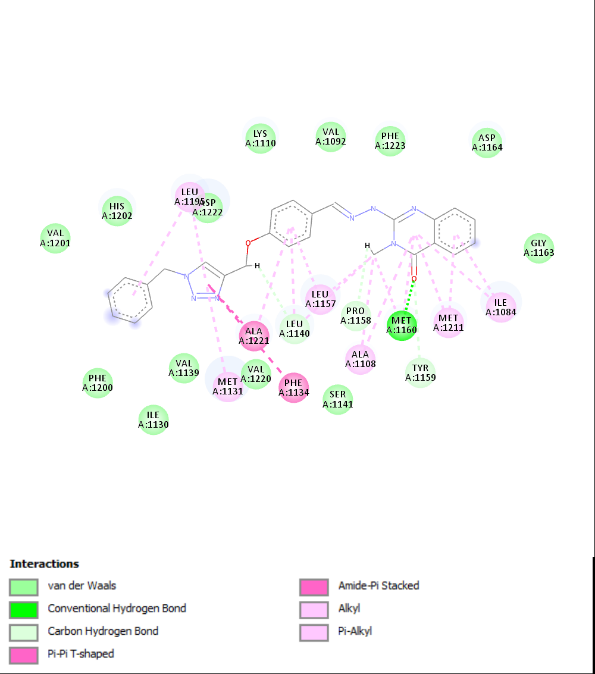 | Cluster 4  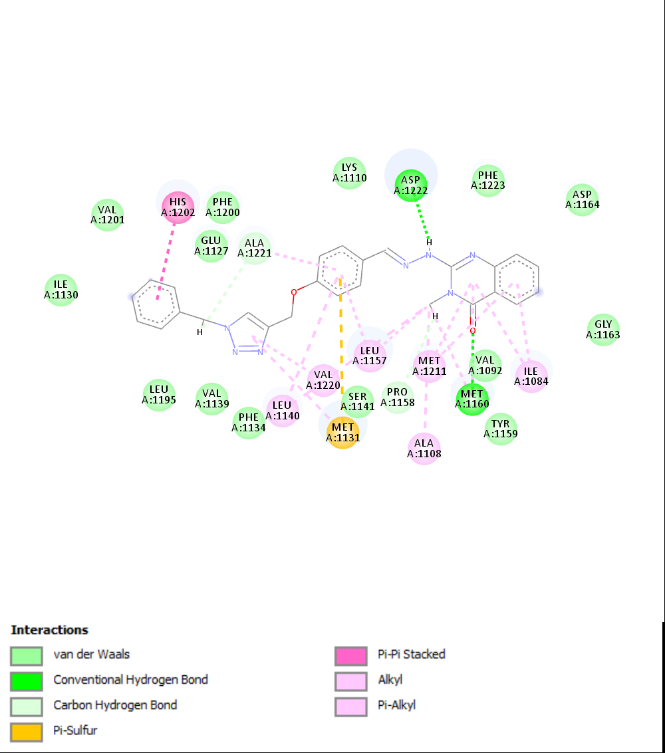 |

**Cluster analysis of CM9 with FLT4 t kinase**

| Cluster 1  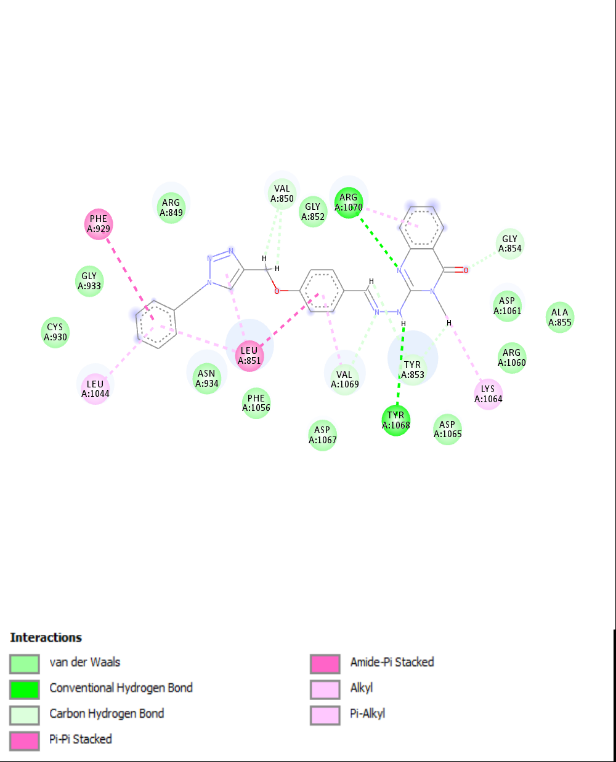 | Cluster 2  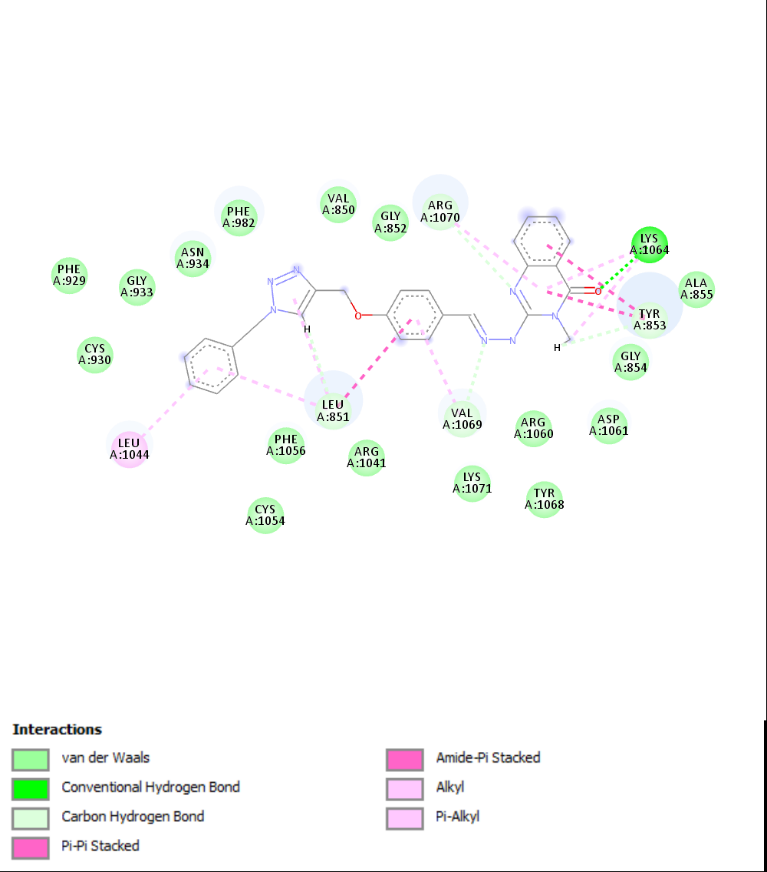 |
| --- | --- |
| Cluster 3  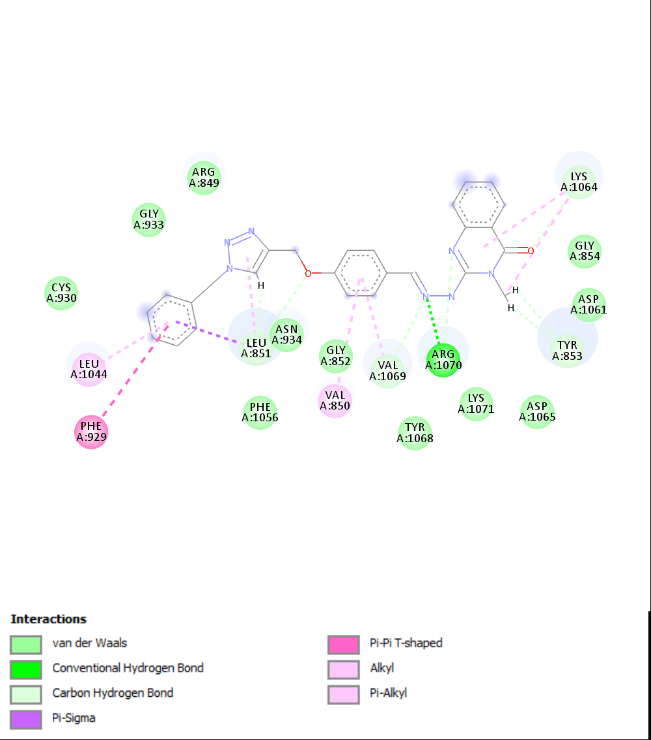 | |
